# Polydatin improves vascular endothelial function by maintaining mitochondrial homeostasis under high glucose conditions

**DOI:** 10.1038/s41598-023-43786-4

**Published:** 2023-10-02

**Authors:** Wahid Shah, Qiyue Zhao, Sen Wang, Miaomiao Zhang, Hongyu Ma, Yue Guan, Yi Zhang, Yan Liu, Chunhua Zhu, Sheng Wang, Xiangjian Zhang, Jinghui Dong, Huijie Ma

**Affiliations:** 1 Department of Physiology, Hebei Medical University, Shijiazhuang, 050017 Hebei China; 2 Hebei Collaborative Innovation Center for Cardio-Cerebrovascular Disease, Shijiazhuang, 050017 Hebei China; 3 Department of Endocrinology, The Third Hospital of Hebei Medical University, Ziqiang Road 139, Shijiazhuang, 050051 Hebei China; 4 Department of Neurology, The Second Hospital of Hebei Medical University, Shijiazhuang, 050000 Hebei China; 5 Key Laboratory of Neurophysiology of Hebei Province, Shijiazhuang, 050017 Hebei China; 6 The Key Laboratory of Neural and Vascular Biology, Ministry of Education, Hebei Medical University, Shijiazhuang, 050017 Hebei China

**Keywords:** Cardiovascular biology, Diabetes, Cardiovascular biology

## Abstract

Previous studies have shown that polydatin (Poly) confer cardioprotective effects. However, its underlying mechanisms remain elusive. This study showed that Poly (10 µM) treatment reversed the high glucose (HG)-induced decrease in acetylcholine-elicited vasodilation in aortas. Poly also improved the acetylcholine-induced vasodilation of aortic vessels isolated from diabetic rats. Meanwhile, Poly ameliorated the morphological damage of the thoracic aorta and improved the viability of HUVECs under HG conditions. Furthermore, analysis of the vasoprotective effect of Poly under HG conditions by transmission electron microscopy, Western blotting, and qPCR revealed that Poly improved endothelial pyroptosis through the NLRP3/Caspase/1-IL-1β pathway, enhanced dynamin-related protein 1-mediated mitochondrial fission, and increased the mitochondrial membrane potential under HG conditions. In conclusion, Poly restored acetylcholine-induced vasodilation impaired by HG incubation, which was associated with reduced oxidation, inflammation, and pyroptosis, the recovery of the mitochondrial membrane potential and maintenance of mitochondrial dynamic homeostasis of endothelial cells in the aortas.

## Introduction

Persistent hyperglycemia in diabetes mellitus (DM) often leads to vascular complications^1^. The endothelium plays a pivotal role in the regulation of vascular tone by synthesizing and releasing an array of endothelium-derived relaxing factors. Hyperglycemia-induced endothelial disorder, characterized by compromised endothelium-dependent relaxation (EDR), is one of the typical pathophysiological mechanisms of vascular complications in DM^2^. Coupled with the combined effects of nitric oxide synthase (NOS) and other inflammatory factors, endothelial dysfunction in hyperglycemia leads to a redox imbalance in cells, which plays a key role in regulating vascular tone and maintaining vascular homeostasis^2,3^. Activation of inflammation subsequently lead to cellular dysfunction or injury via pyroptosis, a type of cell death that combines apoptotic and necrotic features^4^. Increasing evidence suggests that morphological and functional changes in the mitochondria are associated with vascular endothelial dysfunction. Moreover, the mitochondrial dynamic imbalance leads to mitochondrial breakage and disrupts endothelial physiological functions^5^.

Polydatin (Poly) extracted from the roots and rhizome of *Reynoutria japonica* is a widely used traditional Chinese medicine. It is also present in red wine, peanuts, hops pellets, chocolate, cocoa products, and various daily foods^6^. The biomedical properties of Poly include antiplatelet aggregation, antioxidation, cardioprotection, anti-inflammation, and immune regulation^6^. Poly treatment has been shown to normalize glucose and lipid metabolism, reverse insulin resistance, and reduce experimental diabetes-induced fibrosis^7,8^. Furthermore, Poly has been shown to restore EDR in aortic rings impaired by high glucose (HG) via the peroxisome proliferator-activated receptor alpha (PPARβ)/nitric oxide (NO) signaling pathway^9^. However, whether anti-inflammation, anti-pyroptosis, and mitochondrial dynamic homeostasis mediate the vasodilation effect of Poly remains elusive.

Herein, we hypothesize that Poly ameriolates HG-induced impairement of EDR by alleviating inflammation and oxidative stress (OS) and maintaining mitochondrial dynamic homeostasis of endothelial cells (ECs). This study will offer new insights into the study of the vasodilation effect of Poly.

## Results

### Effect of Poly on vasodilation under HG condition

**Figure 1:**
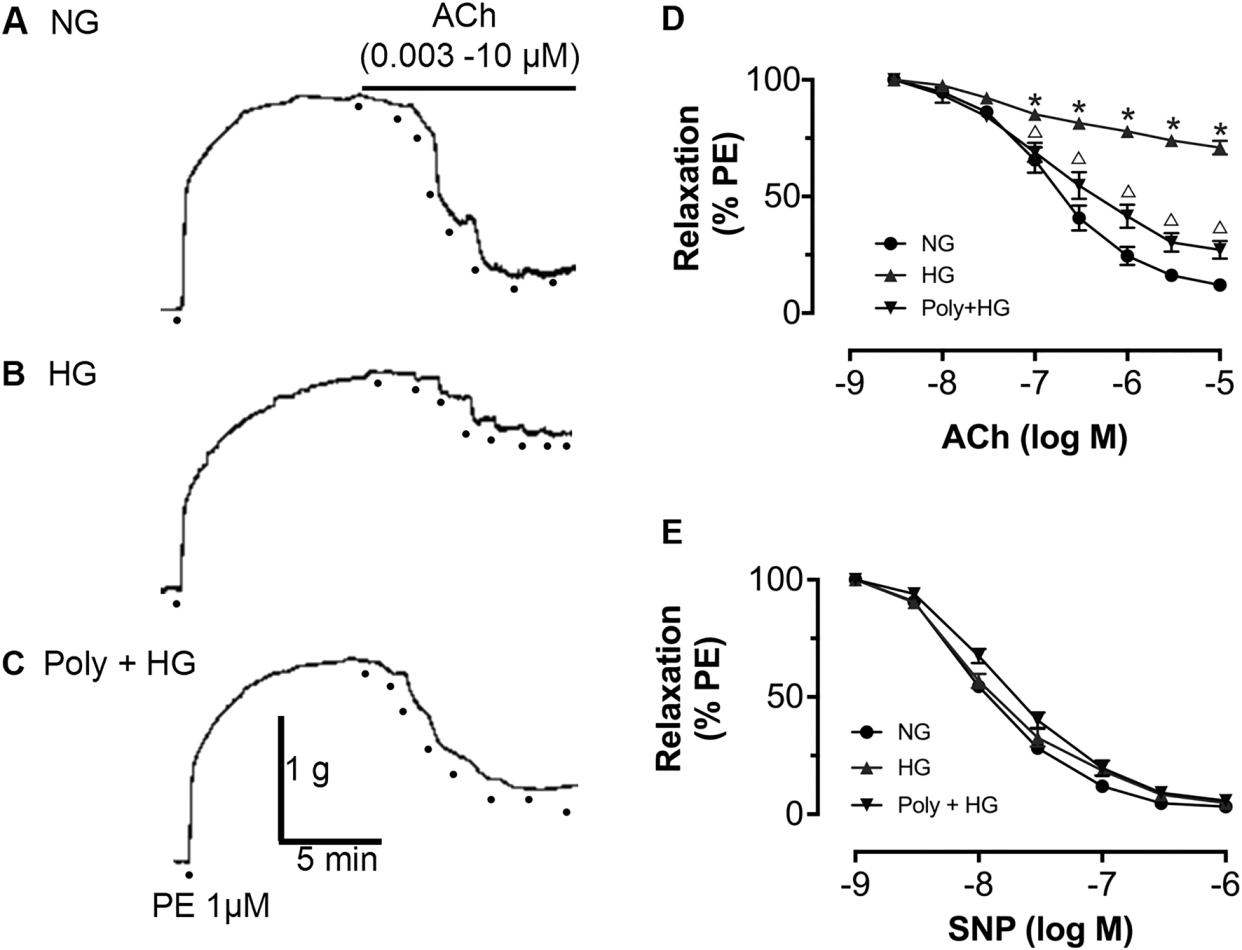
Effect of polydatin on endothelium-dependent vasodilation of ACh under high-glucose condition. ACh (0.003–10 µM)-induced vasodilation responses in the aortas pre-contracted by phenylephrine (1 µM) were significantly impaired by high glucose (HG) incubation. Co-incubation of polydatin (Poly, 10 µM) under HG conditions for 6 h significantly improved endothelium-dependent relaxation of ACh. (**A**–**C**) Typical traces of the dose–response relationship for ACh on rat aortas. (**D**) Dose–response curve of ACh on aortic rings incubated with normal glucose (NG), HG, and Poly + HG (*n* = 6 per group). (**E**) Endothelium-independent sodium nitroprusside-induced relaxations were similar among the groups (*n* = 5). Data are expressed as mean ± SEM. *P < 0.05 versus NG, ^△^*P* < 0.05 versus HG.

After 6 h of incubation with HG, phenylephrine (PE) (1 µM, MedChemExpress, USA)-constricted aortic rings showed similar contractility to the normal glucose (NG) group (*P* > 0.05). Acetylcholine (Ach) (0.003–10 µM, Sigma, USA) caused a dose-dependent relaxation under the NG condition (Fig. 1A,D). ACh-induced vasodilation was significantly reduced in HG-incubated aortic rings (*P* < 0.05, Fig. 1B,D), which indicated impaired EDR. Poly (10 µM, TCI, Japan) improved the impaired ACh-induced vasodilation under HG condition (*P* < 0.05, Fig. 1C,D). In contrast, no significant difference was observed among groups in endothelium-independent vasodilation by SNP (Fig. 1E). In addition, Poly also decreased the acetylcholine-induced vasodilation of aortas isolated from diabetic rats (Supplementary Fig. 1). These results suggest that Poly may partially improve the decrease in endothelial-dependent relaxation in the aortic ring under HG conditions.

### Effect of Poly on aortic ring morphology and viability of HUVECs

**Figure 2:**
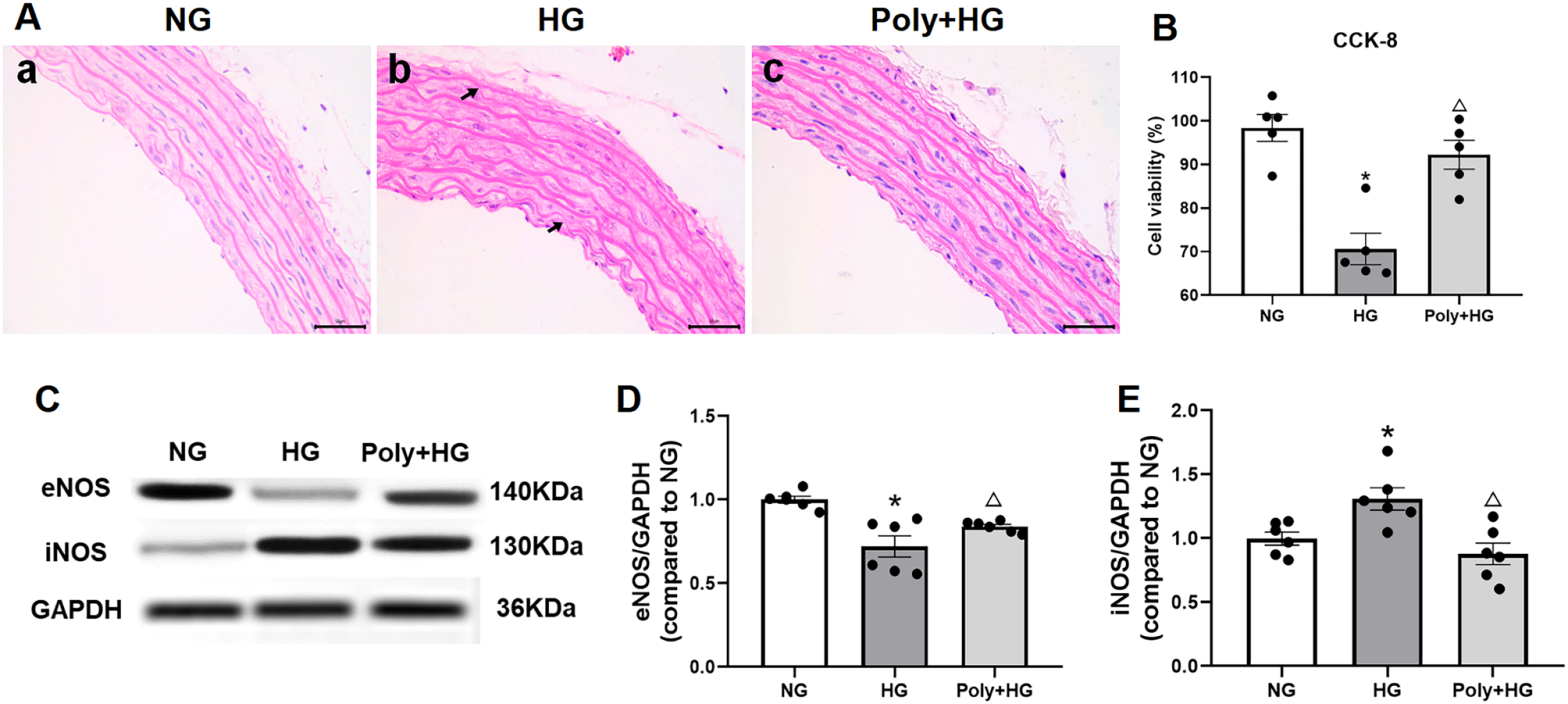
Effect of polydatin on morphological changes of aortic rings and viability of HUVECs under high-glucose condition. (**A**) Representative HE–stained thoracic aortas of normal glucose (NG) incubation (a), high glucose (HG) incubation (b), and polydatin (Poly, 10 µM) co-incubation with HG (Poly + HG) (c). Scale bar: 50 µm. (**B**) CCK-8 assay showed that the viability ratio was increased in HUVECs after Poly treatment under HG conditions (*n* = 5 per group). (**C**–**E**) Representative bands (**C**) and summarized data (**D**, **E**) of eNOS and iNOS protein expression in NG, HG, and Poly + HG groups (*n* = 6 per group). Data are expressed as mean ± SEM. *P < 0.05 versus NG, ^△^*P* < 0.05 versus HG.

Hematoxylin and eosin (HE) staining data showed that the internal elastic lamina was disrupted after incubation for 6 h with HG. However, the aortic damages caused by HG were ameliorated by pretreatment with Poly (Fig. 2A).

To further investigate whether Poly could improve the viability of ECs incubated with HG, the cell counting (CCK-8) assay was performed on HUVECs. Results of the CCK-8 assay showed that the viability ratio of ECs decreased after incubation with HG (30 mM), which was significantly improved by Poly co-incubation with HG (Poly + HG) (*P* < 0.05, Fig. 2B). These results indicated that Poly ameliorated the morphological damages of the thoracic aorta and improved the viability of HUVECs under HG conditions.

### Involvement of NOS in Poly’s action on aortic rings under HG condition

NO is a critical factor that regulates endothelium-dependent vasodilation in the aortas, which was commonly associated with endothelial NO synthase (eNOS)^10^. Our study showed that pre-incubation with L-NAME (100 µM, Sigma, USA), an eNOS inhibitor, completely abolished the amelioration of ACh-induced vasodilation by Poly under HG condition (Supplementary Fig. 2).

OS- or inflammation-induced inducible NOS (iNOS) contributes to severe endothelium injury and EDR^9^. Our results revealed that the expression level of eNOS decreased, while the expression level of iNOS increased significantly after incubation with HG (*P* < 0.05, Fig. 2C–E). However, Poly (10 µM) incubation reversed the expression changes in eNOS and iNOS of aortic rings (*P* < 0.05, Fig. 2C–E). The above results indicate that the impaired vasodilation of aortic rings under HG conditions was ameriolated through a mechanism involving upregulation of eNOS expression and downregulation of iNOS expression.

### Effect of Poly on the free radical level

**Figure 3:**
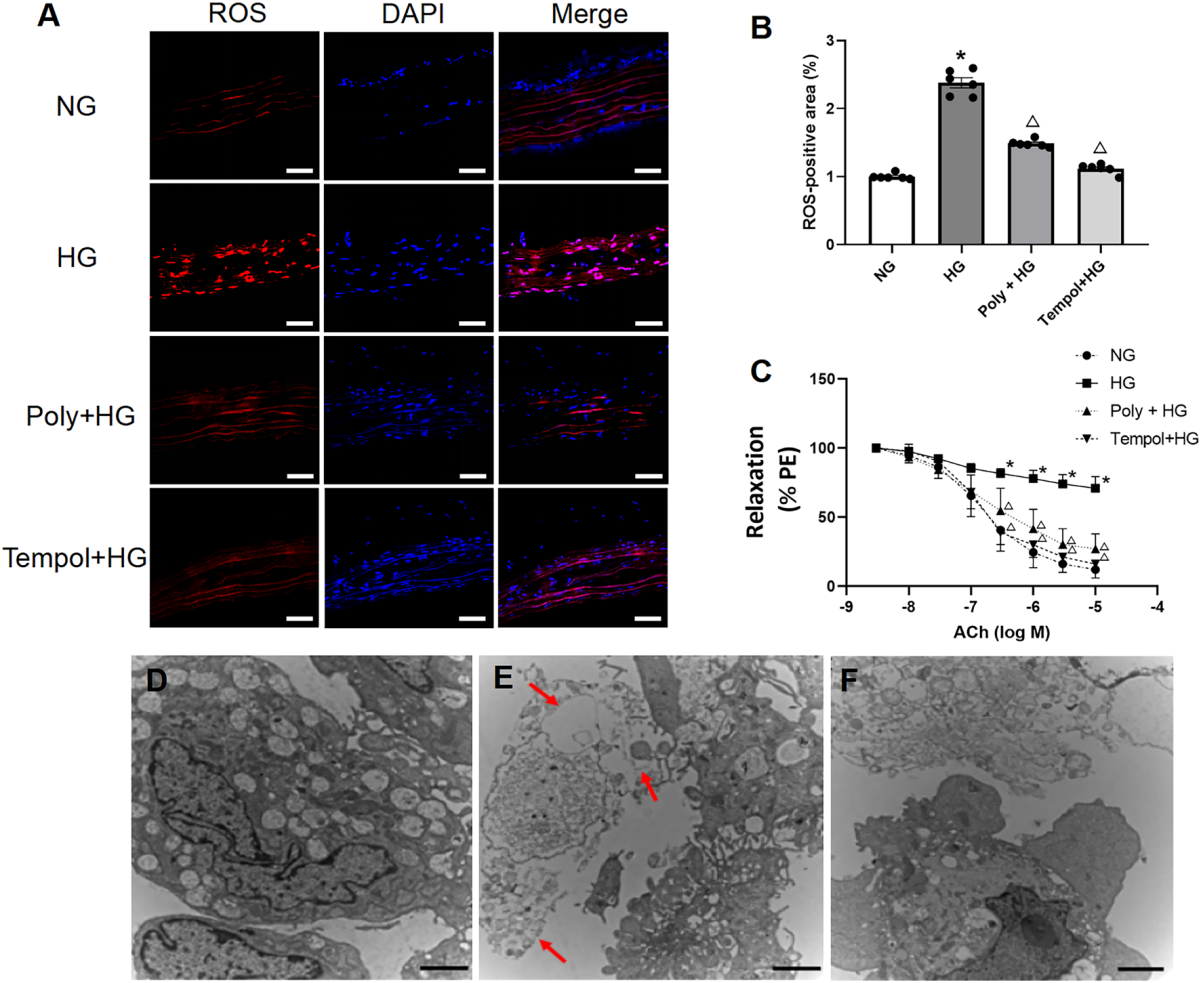
Polydatin reduces free radical levels of thoracic aortas and pyroptosis in HUVECs under high-glucose condition. (**A**, **B**) Polydatin (Poly, 10 µM) and tempol significantly reduced ROS levels of thoracic aortas under high glucose (HG) conditions. Representative images (**A**) and quantified data (**B**) of labeled ROS-positive cells in the thoracic aortas (*n* = 5 rats per group). Scale bar: 50 µm. Data are expressed as means ± SEM. *P < 0.05 versus normal glucose (NG), ^△^*P* < 0.05 versus HG. (**C**) Dose–response curve of ACh on aortic rings incubated with NG, HG, Poly + HG and Tempol + HG (*n* = 6 per group). (**D**–**F**) Pyroptosis of HUVECs detected by TEM in NG (**D**), HG (**E**), and Poly + HG (**F**). Note that pyroptosis occurred in HUVECs incubated with HG, which appeared swollen and with an enlarged size (the upper and middle arrows) and exhibited a necrosis-like pattern characterized by the loss of plasma membrane integrity and the release of cellular contents into the extracellular space (the bottom arrow), and HUVECs incubated with Poly + HG showed almost normal endothelial cells. Scale bar: 2 µm.

To investigate whether increased free radical levels contribute to the impaired relaxation of the aorta, the reactive oxygen species (ROS) level was lowered by a free radical scavenger tempol (100 µM, Sigma, USA). Immunofluorescence staining results showed that HG incubation increased ROS expression, and both the Poly and tempol significantly decreased the elevated ROS expression under HG conditions (*P* < 0.05, Fig. 3A,B). The effect of tempol incubation on ameliorating ACh-induced vasodilation under HG conditions was similar to that of Poly (Fig. 3C). Transmission electron microscopy **(**TEM) results demonstrated that HUVECs exposed to HG exhibited swelling and increased size, suggesting a potential disturbance in cellular balance. Additionally, the TEM images revealed a necrosis-like pattern in HUVECs exposed to HG, marked by compromised plasma membrane integrity and the release of cellular contents into the extracellular space. However, HUVECs incubated with Poly + HG showed almost normal ECs (Fig. 3D–F). These results indicate that Poly exerts a protective effect on the endothelium through anti-OS.

### Effect of Poly on NOD-like receptor thermal protein domain associated protein 3 (NLRP3), vascular cell adhesion molecule 1 (VCAM-1), caspase-1 (CSP-1), and interleukin-1 beta (IL-1β) expression in aortic rings and cultured HUVECs under HG condition

**Figure 4:**
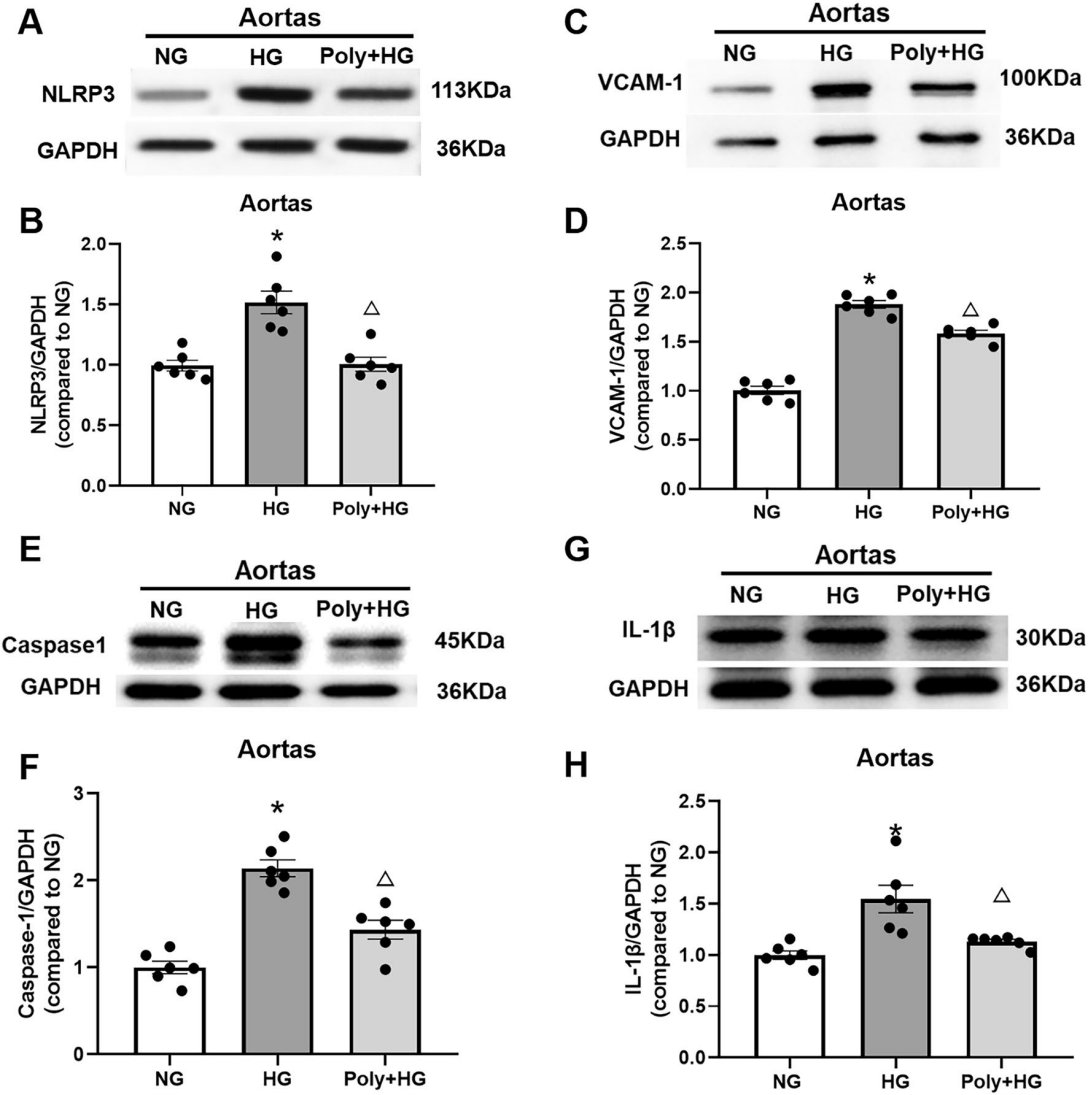
Effect of polydatin on protein expression level of NLRP3, VCAM-1, CSP-1, and IL-1β in the aortas under high-glucose condition. (**A**, **B**) Representative bands (**A**) and summarized data (**B**) of NLRP3 protein expressions in the aortas of normal glucose (NG), high glucose (HG), and polydatin (Poly, 10 µM) + HG groups. (**C**, **D**) Representative bands (**C**) and summarized data (**D**) of VCAM-1 protein expression in the aortas of NG, HG, and Poly + HG groups. (**E**, **F**) Representative bands (**E**) and summarized data (**F**) of CSP-1 protein expression in the aortas of NG, HG, and Poly + HG groups. (**G**–**H**) Representative bands (**G**) and summarized data (**H**) of IL-1β protein expression in the aortas of NG, HG, and Poly + HG groups. Data are expressed as mean ± SEM; *n* = 6 per group. *P < 0.05 versus NG, ^△^*P* < 0.05 versus HG.

To specifically determine whether Poly could reduce inflammation under HG conditions, we measured the protein levels of NLRP3, VCAM-1, CSP-1, and IL-1 β of the aortic ring in each group. As shown in Fig. 4, the protein levels of NLRP3, VCAM-1, CSP-1, and IL-1 β were increased following HG exposure (*P* < 0.05), which were reversed by pretreatment with Poly (10 µM) (*P* < 0.05, Fig. 4A–H).

**Figure 5:**
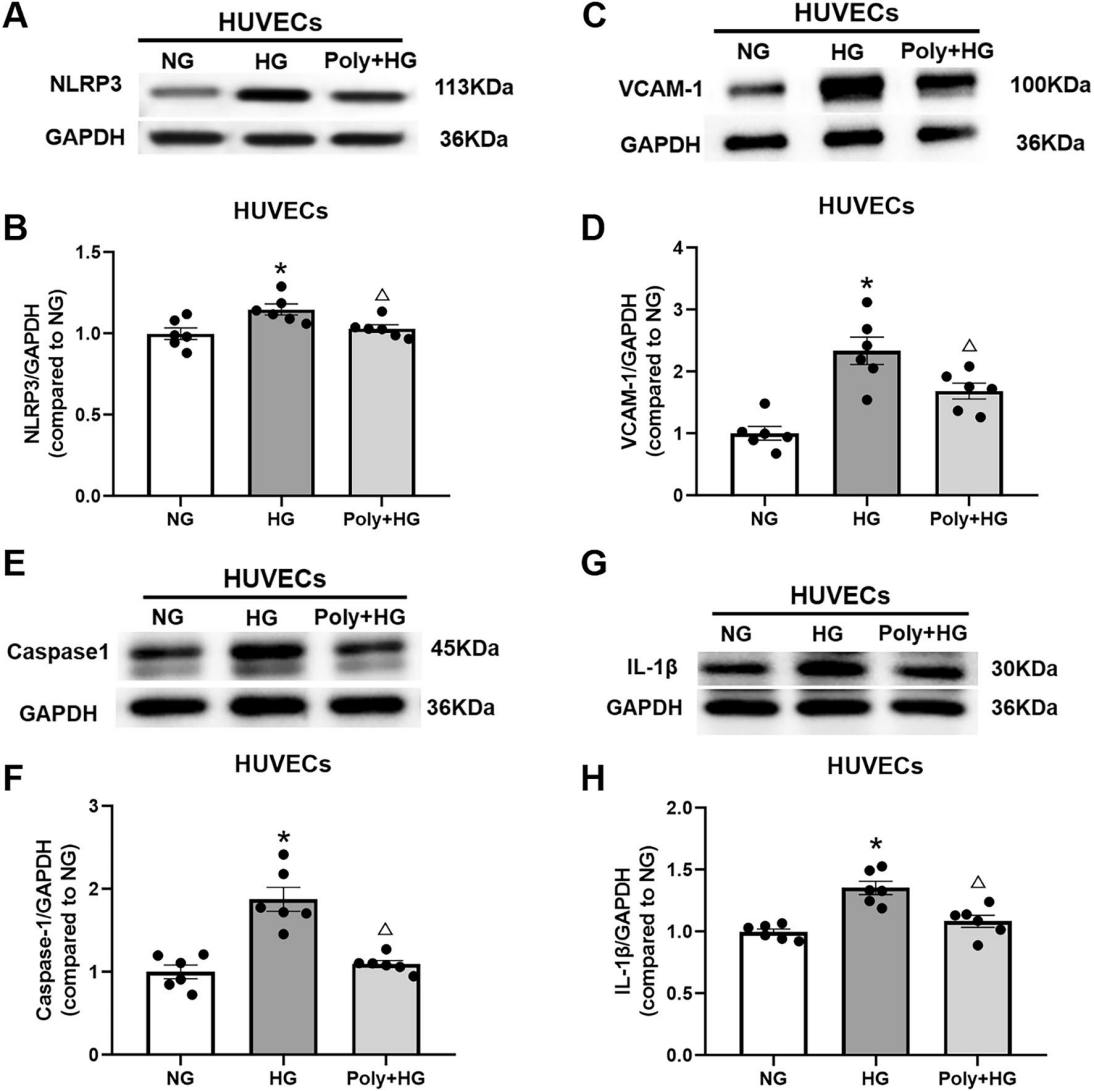
Effect of polydatin on protein expression level of NLRP3, VCAM-1, CSP-1, and IL-1β in HUVECs under high-glucose condition. (**A**, **B**) Representative bands (**A**) and summarized data (**B**) of NLRP3 protein expressions in HUVECs of normal glucose (NG), high glucose (HG), and polydatin (Poly, 10 µM) + HG groups. (**C**, **D**) Representative bands (**C**) and summarized data (**D**) of VCAM-1 protein expression of HUVECs in the NG, HG, and Poly + HG groups. (**E**, **F**) Representative bands (**E**) and summarized data (**F**) of CSP-1 protein expression in HUVECs of NG, HG, and Poly + HG groups. (**G**, **H**) Representative bands (**G**) and summarized data (**H**) of IL-1β protein expression in HUVECs of NG, HG, and Poly + HG groups. Data are expressed as mean ± SEM; *n* = 6 per group. *P < 0.05 versus NG, ^△^*P* < 0.05 versus HG.

HG treatment also increased protein levels of NLRP3, VCAM-1, CSP-1, and IL-1 β in HUVECs. Poly (10 µM) co-incubation with HG reversed the increased protein levels of NLRP3, VCAM-1, CSP-1, and IL-1β in HUVECs (*P* < 0.05, Fig. 5A–H). These data suggest that Poly improves inflammation and pyroptosis of endothelium through the NLRP3/CSP-1/IL-1β signaling pathway.

### Effect of Poly on the mitochondrial membrane potential (MMP) of cultured HUVECs under HG condition

**Figure 6:**
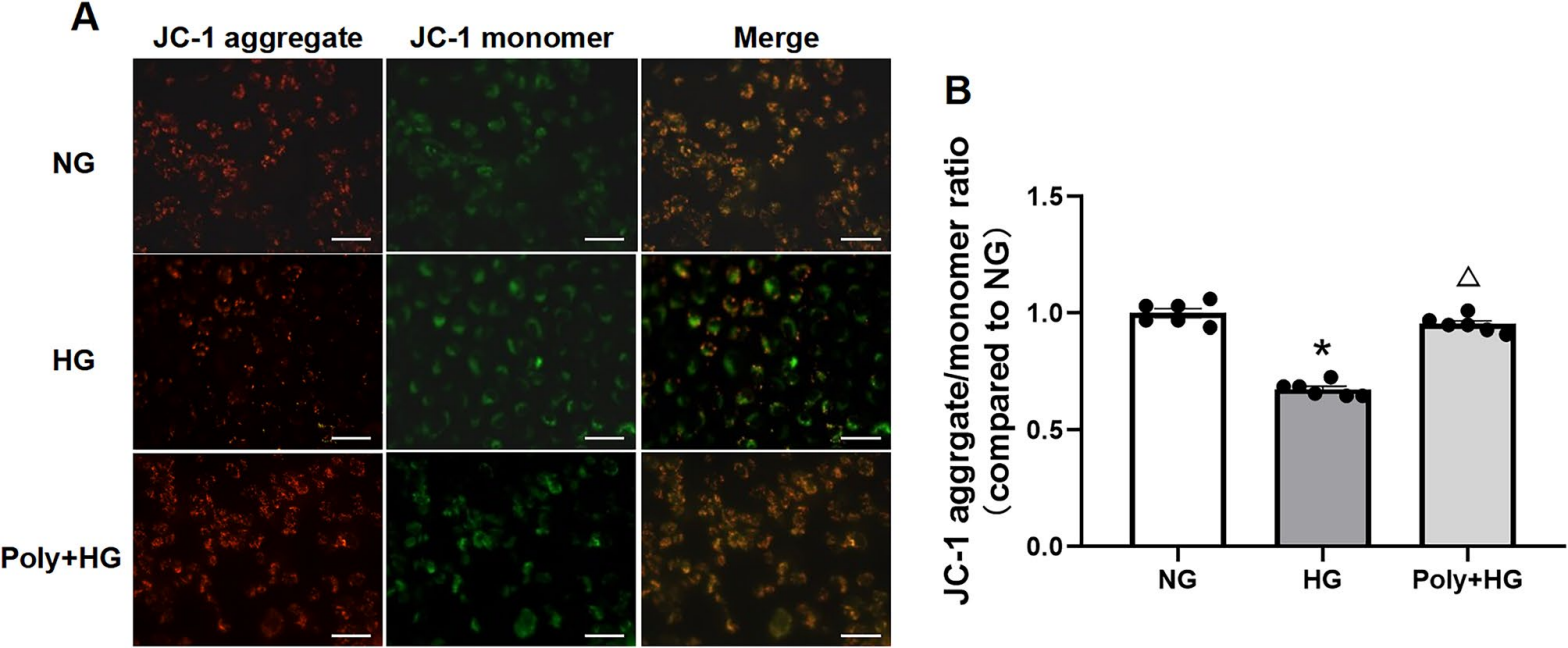
Effect of polydatin on mitochondrial membrane potential (MMP) in HUVECs. (**A**) Representative images showing JC-1 aggregates (red) and JC-1 monomers (green) in HUVECs incubated with normal glucose (NG), high glucose (HG), and polydatin (Poly, 10 µM) + HG groups measured by JC-1 staining. (**B**) MMP statistic data showing the JC-1 aggregate/monomer ratio changes in NG, HG, and Poly + HG group. Note that the higher the red/green fluorescence ratio of the dye in the mitochondria, the higher the MMP. Scale bar: 50 µm. Data are expressed as mean ± SEM; *n* = 6 per group. *P < 0.05 versus NG, ^△^*P* < 0.05 versis HG.

ROS in cells mainly comes from the mitochondria^11^. The MMP plays a key role in mitochondrial homeostasis, and a decrease or increase of MMP in cells may lead to cell viability loss and is a factor in various diseases^12^. A fluorescent probe 5,5',6,6'-tetrachloro-1,1',3,3'-tetraethylbenzimidazolylcarbocyanine iodide (JC-1) was used to test the effect of Poly on MMP. Results showed that the JC-1 aggregate/monomer ratio was decreased after incubation with HG, which was reversed after Poly co-incubation with HG (*P* < 0.05, Fig. 6). The above results suggest that Poly restored the decreased MMP of HUVECs under HG conditions.

### Effect of Poly on mitochondrial dynamic homeostasis in aortic rings and cultured HUVECs under HG condition

**Figure 7:**
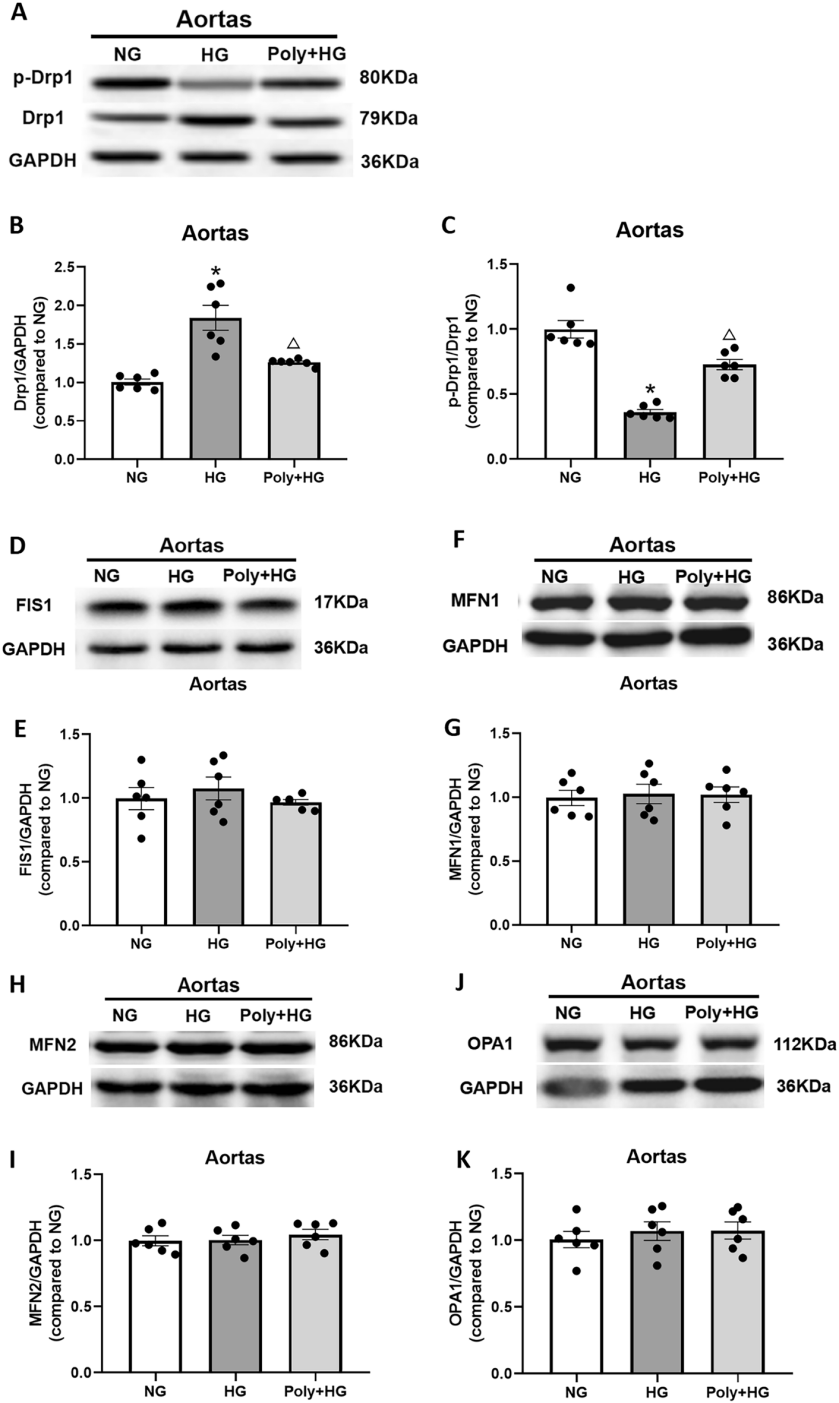
Effect of polydatin on protein expression of Drp1, p-Drp1, FIS1, MFN1, MFN2, and OPA1 in the aortas under high-glucose condition. (**A**–**C**) Representative bands (**A**) and summarized data (**B**, **C**) of Drp1 and p-Drp1 (Ser 637) levels and the p-Drp1/Drp1 ratio in the aortas of normal glucose (NG), high glucose (HG), and polydatin (Poly, 10 µM) + HG groups. (**D**, **E**) Representative bands (**D**) and summarized data (**E**) of FIS1 protein expression in the aortas of NG, HG, and Poly + HG groups. (**F**, **G**) Representative bands (**F**) and summarized data (**G**) of MFN1 protein expression in the aortas of NG, HG, and Poly + HG groups. (**H**, **I**) Representative bands (**H**) and summarized data (**I**) of MFN2 protein expression in the aortas of NG, HG, and Poly + HG groups. (**J**, **K**) Representative bands (**J**) and summarized data (**K**) of OPA1 protein expression in the aortas of NG, HG, and Poly + HG groups. Data are expressed as mean ± SEM; *n* = 6 per group. * P < 0.05 versus NG, ^△^*P* < 0.05 versus HG.

**Figure 8:**
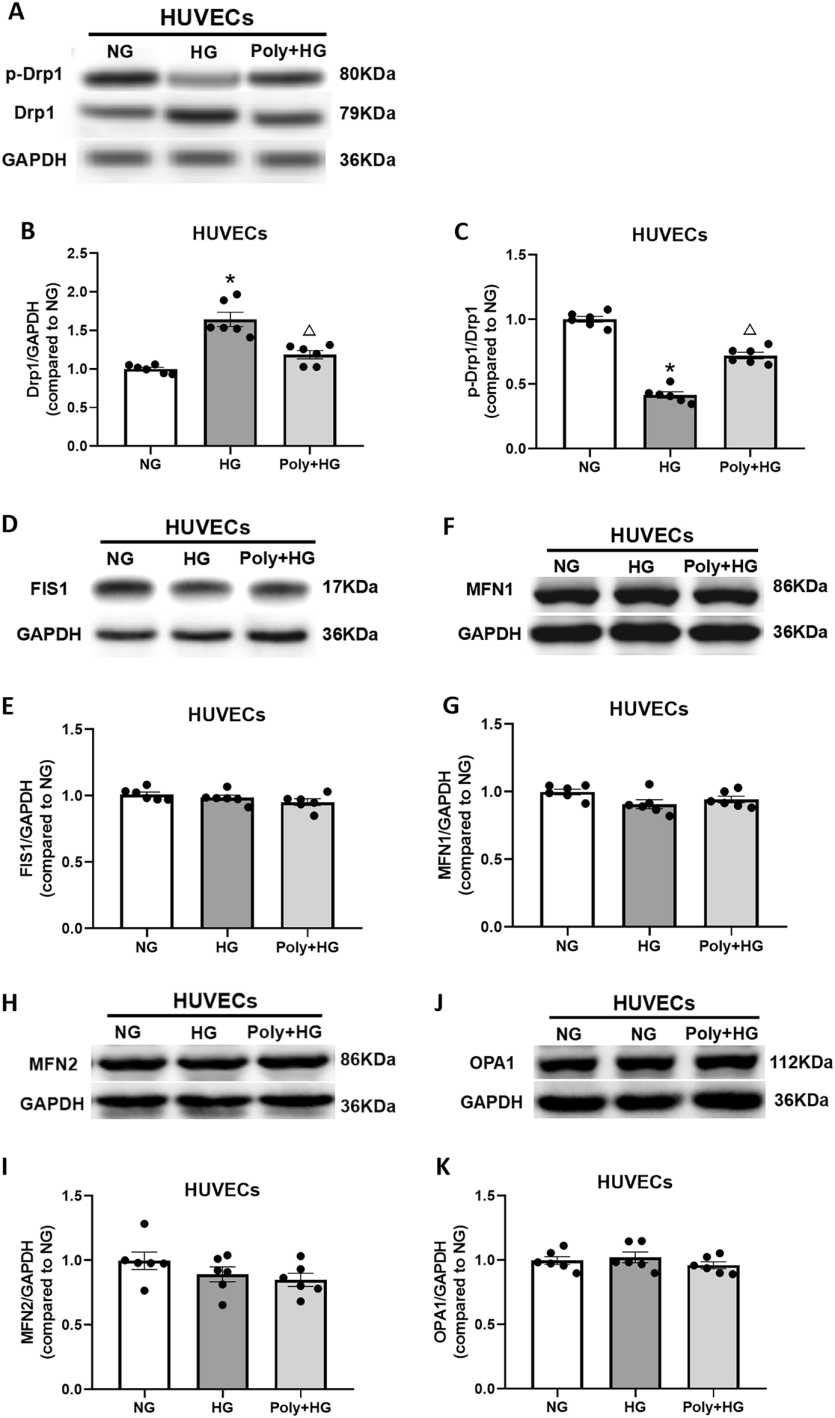
Effect of polydatin on protein expression of Drp1, p-Drp1, FIS1, MFN1, MFN2, and OPA1 in HUVECs under high-glucose condition. (**A**–**C**) Representative bands (**A**) and summarized data (**B**, **C**) of Drp1 and p-Drp1 (Ser 637) levels and the p-Drp1/Drp1 ratio in HUVECs of normal glucose (NG), high glucose (HG), and polydatin (Poly, 10 µM) + HG groups. (**D**, **E**) Representative bands (**D**) and summarized data (**E**) of FIS1 protein expression in HUVECs of NG, HG, and Poly + HG groups. (**F**, **G**) Representative bands (**F**) and summarized data (**G**) of MFN1 protein expression in HUVECs of NG, HG, and Poly + HG groups. (**H**, **I**) Representative bands (**H**) and summarized data (**I**) of MFN2 protein expression in HUVECs of NG, HG, and Poly + HG groups. (**J**, **K**) Representative bands (**J**) and summarized data (**K**) of OPA1 protein expression in HUVECs of NG, HG, and Poly + HG groups. Data are expressed as mean ± SEM; *n* = 6 per group. *P < 0.05 versus NG, ^△^*P* < 0.05 versus HG.

Dynamin-related protein-1 (Drp1), which regulates mitochondrial fission, is also involved in the maintenance of mitochondrial homeostasis. Drp1 overexpression resulted in abundant mitochondrial fission, ROS accumulation, and loss of MMP^13,14^. To examine whether Poly could modulate mitochondrial dynamic homeostasis, we measured the protein levels of total Drp1 and phosphorylated Drp1 (p-Drp1, Ser637) in aortas and HUVECs. The level of Drp1 protein was increased significantly in the aorta and HUVECs after HG incubation compared with NG incubation (*P* < 0.05). However, Poly pretreatment significantly reversed this effect (*P* < 0.05, Figs. 7A,B and 8A,B). The protein levels of p-Drp1 (Ser637) were markedly reduced (*P* < 0.05), while the application of Poly significantly increased the levels of p-Drp1 (Ser637) in the aortas (*P* < 0.05, Supplementary Fig. 3A) and HUVECs (*P* < 0.05, Supplementary Fig. 3B). Furthermore, the p-Drp1/Drp1 ratio was significantly lower in the HG group relative to the NG group and increased in the Poly + HG group compared with the HG group in the aortas (*P* < 0.05, Fig. 7C) and HUVECs (*P* < 0.05, Fig. 8C).

Mitochondrial fission protein 1 (FIS1) plays a central part in mitochondrial fission^15^. We tested the protein levels of FIS1 in the aortas and HUVECs. No significant differences in FIS1 protein expressions in the aortas (*P* > 0.05, Fig. 7D,E) and HUVECs (*P* > 0.05, Fig. 8D,E) were found among NG, HG, and Poly + HG groups.

Integral membrane proteins mitofusin 1 (MFN1) and mitofusin 2 (MFN2) mediate the fusion of adjacent mitochondrial outer membranes, while optic atrophy 1 (OPA1) mediates the inner membrane fusion and cristae remodeling^15^. To explore whether Poly affects the mitochondrial fusion of ECs, we measured the protein expression level of MFN1, MFN2, and OPA1 in the aortas and HUVECs. The results showed that neither HG nor Poly affected the protein expressions of MFN1, MFN2, and OPA1 in the aortas (*P* > 0.05, Fig. 7F–K) and HUVECs (*P* > 0.05, Fig. 8F–K). These results indicate that Poly protects aortas and HUVECs from HG injury by improving Drp1-mediated mitochondrial fission.

## Discussion

The present study investigated whether Poly can ameliorate the vasodilation effects HG on rat aortic rings and the potential underlying mechanisms. The results showed that Poly effectively attenuated endothelium-dependent vasodilation induced by HG, which was abolished by the eNOS inhibitor L-NAME. In addition, Poly alleviated HG-induced morphological impairment of the aortic ring, increased the expression of eNOS, and decreased the expression of iNOS under HG conditions. Meanwhile, Poly decreased the elevated ROS level, inflammatory response, and pyroptosis. Furthermore, Poly recovered the decreased MMP and p-Drp1 (Ser637) levels but increased the total Drp1 levels under HG conditions in both the aortas and HUVECs. Overall, our study suggested that Poly restores endothelial-induced vasodilation by alleviating OS, inflammation, and pyroptosis and improving mitochondrial function and dynamics under HG conditions.

NOS is expressed in the nervous system and consists of three isoforms of its isoenzymes: neuronal nitric oxide synthase (nNOS) and eNOS, which are expressed in normal conditions, and iNOS, which is expressed following injury. NO derived from eNOS had endothelial protective effects^9,16^. However, NO derived from iNOS and nNOS had endothelial toxic effects^17^. Consistent with previous studies^9^, our results showed that Poly reversed the adverse effects of HG on ACh-induced vasodilation in aortic rings and the cell viability of HUVECs. The enhanced EDR of Poly under HG condition was obstructed by the eNOS inhibitor L-NAME. We also demonstrated that HG resulted in a marked decrease in eNOS protein levels and an increase in iNOS protein levels in the aortas. However, pretreatment with Poly reversed the protein expression changes in eNOS and iNOS. Meanwhile, Poly also improved the morphological injury of aortic rings induced by HG incubation. These results suggested that the eNOS-NO system is involved in the ameliorative effect of Poly on impaired EDR caused by HG.

Inflammation is one of the factors that trigger endothelial dysfunction in the pathophysiology of diabetic macrovascular disorders. Several studies have confirmed the anti-inflammatory activity of Poly in the prevention of heart and neurodegenerative diseases^6^, steatohepatitis^18^, fructose-induced liver inflammation^19^, and adipose tissue inflammation^20^. NLRP3 is a member of the nucleotide-binding domain-(NOD)-like receptor family^21^, and NLRP3 inflammasome-mediated inflammation and pyroptosis play a key role in vascular endothelial dysfunction^22^. Previous studies showed that the NLRP3 inflammasome promotes endothelial inflammation under HG conditions^23,24^. In addition, VCAM-1 is expressed in ECs and mediates leukocyte adhesion in response to cytokines such as tumor necrosis factor-alpha and IL-1β^25–27^, and further activates endothelial signals to induce vascular damage^28^. We showed that Poly (10 µM) reversed the increased protein expressions of NLRP3, VCAM-1, IL-1β, and CSP-1 in aortic rings and HUVECs after HG incubation. Our results indicate that Poly may alleviate the inflammatory response in the endothelium of aortic rings and HUVECs under HG conditions.

Pyroptosis is a type of inflammatory cell death that combines apoptotic and necrotic characteristics and is mediated by pyroptotic caspases, such as CSP-1 activation^29^, which is responsible for the cleavage of IL-1β and IL-18. It has been shown that pyroptosis plays an essential role in cardiovascular diseases^30,31^, diabetic heart disease^32^, and cardiac ischemia–reperfusion injury^33^. The caspase-cascade system is involved in the initiation, transduction, and amplification of tissue and cell apoptosis^34^. Our TEM results showed that Poly alleviated the disruption of HUVECs induced by HG. We hypothesize that Poly might attenuate pyroptosis of HUVECs exposed to HG. Collectively, the findings of this study indicate that Poly can improve endothelial pyroptosis induced by HG via the NLRP3/CSP-1/IL-1β signaling pathway.

ROS are generated as by-products of cellular metabolism, primarily in the mitochondria^35–37^. When the electron transport chain on the inner mitochondrial membrane is disrupted by hyperglycemia in diabetes, ROS accumulate in the cell and become toxic after reaching a certain level^30,38^, which leads to mitochondrial dysfunction^39,40^. In addition to being a primary source of ROS, mitochondria are also the targets of ROS. The MMP plays a key role in mitochondrial homeostasis and a decrease in MMP in the cell may reduce cell viability loss^12^. We showed that the MMP was decreased after HG incubation, whereas this effect was improved by Poly co-incubation with HG. Excessive ROS damages mitochondrial proteins, messenger RNA, and membrane lipids and decreases the MMP, resulting in endothelial dysfunction^41–45^, which could be rescued by Poly.

The dynamic transition of the mitochondria that coordinates fission and fusion cycles plays a vital role in mitochondrial homeostasis^46^. Mitochondrial fission was regulated by the recruitment of the GTPase Drp1 and overexpression of Drp1 resulted in excessive mitochondrial fission, ROS accumulation, and loss of MMP^13,14^. Conversely, phosphorylation of Drp1 at Ser637 inhibited mitochondrial fission^47^. As another integral membrane protein, FIS1 participates in mitochondrial fission. It plays an important role in the regulation of mitochondrial fission as a pro-apoptotic factor^48^. It interacts with Drp1 and facilitates the functional fission complex formation, thereby inducing apoptosis. Our study found that the expression of Drp1 increased in the presence of HG, whereas Ser637 phosphorylation of Drp1 and the p-Drp1/Drp1 ratio was notably decreased in both the aortas and HUVECs. However, Poly significantly decreased these changes under HG conditions. Moreover, neither HG nor Poly affected the expression of FIS1 in the aortas and HUVECs. Overall, these changes were due to a decline in mitochondrial fission and fragmentation associated with hyperglycemia. Our data showed that hyperglycemia induced an increase in Drp1 and a decrease in p-Drp1 (Ser637) in the aortas and HUVECs, which was reversed by Poly. We also found that neither HG nor Poly affected mitochondrial fusion-related protein expressions of MFN1, MFN2, and OPA1 in the aortas and HUVECs, implying that Poly does not affect mitochondrial fusion under HG conditions.

One limitation of this study is that it was conducted solely in animals and ex vivo. Although the results in HUVECs and aortas of the diabetic rat model provide important insights, the applicability of these findings in clinical settings is limited. Further validation is required to ascertain the impact of Poly on chronic hyperglycemia, especially in diabetic patients. Moreover, this study primarily centered on the broad-spectrum effect of Poly on vascular endothelial cells, without delving into the specific upstream and downstream signaling pathways. Therefore, the mechanistic insights are correlative, and additional investigations are needed to elucidate the precise mechanisms by which Poly modulates inflammation, oxidative stress, and mitochondrial function.

## Conclusion

In summary, Poly restores ACh-induced vasodilation impaired by HG incubation, which might be associated with a reduction in the OS, inflammation, and pyroptosis, recovery of MMP, and improvement of mitochondrial dynamics of aortic ECs.

## Experimental protocol

### Experimental animals

Thirteen-week-old male adult Sprague–Dawley rats were purchased from Beijing Vital River Laboratory Animal Technology Co., Ltd. (Beijing, China). Rats were kept in a constant temperature room with an artificially controlled light/dark cycle (12/12 h), with free access to food and water. To establish the DM animal model, 6 rats were randomly selected to intraperitoneally injected with streptozotocin (STZ) (60 mg/kg/d in 0.1 mM sodium citrate buffer; Sigma, St. Louis, MO, USA) for 7 days. The experiments were carried out followed the National Research Council’s (NRC) Guide for the Care and Use of Laboratory Animals and the ARRIVE 2.0 guideline^49^, and all techniques and procedures were approved by the Hebei Medical University Ethics Committee for the Use of Laboratory Animals.

### Preparation of isolated thoracic aortic rings

Rats were anesthetized with 3% isoflurane inhalation. Then rats’ thoracic aortas (3–4 mm long) were dissected and randomly divided into three groups: NG group, HG group, and Poly + HG group. Poly was dissolved in 0.5% dimethysulfoxide (DMSO) and directly diluted in the medium to a concentration of 10 µM for further experiments. The concentration of Poly was determined based on the literatures^50,51^ and our preliminary experiment (Supplementary Figs. 4 and 5). All vascular tissues were carefully separated from connective tissues and placed in organ bath chambers filled with the Krebs–Henseleit (K-H) buffer saturated with 95% oxygen and 5% carbon dioxide (CO_2_) at 37 °C. The K-H buffer consisted of (in mM): 118 NaCl, 4.7 KCl, 2.5 CaCl_2_, 1.2 MgSO_4_, 25.0 NaHCO_3_, 1.2 KH_2_PO_4_, and 11.0 glucose (pH 7.35–7.4).

### Measurement of vascular tone

An organ bath system (Chengdu Instrument Company, Chengdu, China) was used to record the vascular tension. Aortic rings were normalized with an optimal pre-tension and stabilized for 30 min. The contraction of the aortic ring was induced with KCl (60 mM) and PE (1 µM), respectively. The relaxation of the aortic rings was induced by ACh (0.003–10 µM) or sodium nitroprusside (SNP, 0.001–1 µM). ECs were considered intact and data were included if ACh-induced vasodilation in the aortic rings exceeded 80% of PE (1 µM)-induced constriction. Data were excluded if the diastole of the aortic rings was less than 80%. Aortic rings were incubated in K-H solution containing NG (11 mM), HG (55 mM), or Poly (10 µM) + HG (55 mM) for 6 h. In another set of experiments, aortic rings were co-incubated with endothelial NOS (eNOS) inhibitor L-NAME (100 µM) or ROS scavenger tempol (100 µM) for 6 h together with Poly + HG solution. Vessel rings were rinsed with the K-H buffer to remove the effect of the drugs and allow restoration of the aortic tension.

### Histological observation

Aortic tissues were fixed with 4% paraformaldehyde and then embedded in paraffin. The tissue was cut using a microtome into sections with a thickness of 4 µm. The paraffin was then removed and the sections were stained with H&E and observed under a light microscope.

### CCK-8 assay

Human umbilical vein endothelial cells (HUVECs) were supplied by ScienCell Research Laboratories and maintained in an endothelial cell medium (ECM, No. 1001, ScienCell, USA) supplemented with 10% fetal bovine serum (Gibco), 100 U/mL penicillin, and 100 U/mL streptomycin. HUVECs were treated with normal medium (NG), 30 mM glucose medium (HG), and 10 µM Poly + 30 mM glucose medium (Poly + HG) for 48 h. HUVECs were incubated in a humidified incubator with 5% CO_2_ at 37 °C.

Cell viability was measured using the CCK-8 assay (APExBIO, Houston, USA). Cells were seeded in 96-well plates at 5 × 10^3^ cells per well. Then, cells were incubated with 10 µL CCK-8 solution for another 2 h. The absorbance was detected at 450 nm using a microplate reader (Autobio Diagnostics Co, Ltd., Zhengzhou, China).

### ROS measurement

Aortic tissues were embedded in optimum cutting temperature compound (OCT, Sakura, Japan), sliced into 8-µm sections using a cryostat microtome, and stored at − 20 °C after labeling. Frozen slides were returned to room temperature. Next, the slides were washed for 10 min and stained with ROS staining solution (Sigma, USA) after quenching. Slides were observed under fluorescent microscopy and the images were obtained.

### Observation of the cellular ultrastructure under TEM

After fixing with glutaraldehyde, specimens were gradient dehydrated with ethanol and then transferred to acetone (Sigma-Aldrich, MOUSA) for 15 min. Specimens were then incubated with absolute acetone and a final EPON resin mixture overnight. Next, specimens were embedded in a medium in a capsule for 48 h. Finally, sections were stained and observed under Hitachi TEM at 7000 × magnification.

### MMP measurement

The MMP of ECs were analyzed using JC-1 fluorescent probe (No. G1515-100T, Servicebio). Cells in different groups were rinsed with a JC-1 washing buffer and incubated with JC-1 (10 µM) at 37 °C for 30 min. Afterward, cells were rinsed with a washing buffer. The MMP was examined under a fluorescence microscope (Leica, DM6000/DFC310FX). Fluorescence wavelength parameters were adjusted to an excitation wavelength (Ex) of 490 nm and emission wavelength (Em) of 525 nm to detect the JC-1 monomer, and an Ex of 525 nm and Em of 590 nm to detect the JC-1 aggregate. The MMP was represented by a fluorescence ratio of JC-1 aggregate/monomer (red/green). The value of the NG group was set as the standard to normalize the value of the HG and Poly + HG groups.

### Western blotting

The effect of the Poly on protein expression of eNOS, inducible NOS (iNOS), NLRP3, VCAM-1, CSP-1, IL-1β, Drp1, p-Drp1, FIS1, MFN1, MFN2, and OPA1 in the aortas and HUVECs were evaluated using western blotting. Aortic tissues and HUVECs were homogenized on ice in RIPA lysis/extraction buffer (No. BB-3201, BestBio) containing protease and phosphatase inhibitors (No. RP-WA0103, Reportbio; No. HY-K0010, MCE) for 30 min. Samples were centrifuged at 16,000 g for 15 min at 4 °C and the supernatant was obtained. About 50 µg of protein from each sample was separated by 10% sodium dodecyl-sulfate polyacrylamide gel electrophoresis and transferred to a polyvinylidene difluoride membrane. The membranes were incubated with the following primary antibodies: mouse monoclonal anti-eNOS (1:1000, No. 610296, BD Biosciences), rabbit polyclonal anti-iNOS (1:1000, No. ARG56509, Arigobio), rabbit monoclonal anti-NLRP3 (1:1000, No. ET1610-93, HUABIO), mouse monoclonal anti-VCAM-1 (1:1000, No. 66294-1-lg, Proteintech), rabbit polyclonal anti-CSP-1 (1:1000, No. 22915-1-AP, Proteintech), rabbit polyclonal anti-IL-1β (1:1000, No. 16806-1-AP, Proteintech), rabbit polyclonal anti-Drp1 (1:1000, No. 12957-1-AP, Proteintech), rabbit polyclonal anti-pDrp1 (1:1000, No. 3455S, Cell Signal), rabbit polyclonal anti-FIS1 (1:1000, No. 10956-1-AP, Proteintech), rabbit polyclonal anti-MFN1 (1:1000, No. 13798-1-AP, Proteintech), rabbit polyclonal anti-MFN2 (1:1000, No. 12186-1-AP, Proteintech), rabbit polyclonal anti-OPA1 (1:1000, No. 27733-1-AP, Proteintech), and rabbit polyclonal anti-GAPDH (1:1000, No. 60004-1-lg, Proteintech) overnight at 4 °C. Protein bands were detected using an Immobilon™ Western Chemiluminescent HRP Substrate (1:10,000, No. S1001-100 and No. S1002, SeraCare), and images were visualized on a ChemiScope S6 imaging system (CLiNX, China).

### Statistical analysis

Data were expressed as means ± SEM. Statistical analyses were performed using Prism 8 (GraphPad Software Inc., La Jolla, CA). One-way or two-way ANOVA followed by Tukey’s test was used to compare the groups. *P* < 0.05 was considered statistically significant.

### Ethical approval

The experiments were carried out in accordance with the NRC Guide for the Care and Use of Laboratory Animals, and all techniques and procedures were approved by the Hebei Medical University Ethics Committee for the Use of Laboratory Animals.

### Supplementary Information

Supplementary Information 1.

Supplementary Information 2.

## Data Availability

Data are available on request from the corresponding author.
