# Supplementary material for: Polydatin improves vascular endothelial function by maintaining mitochondrial homeostasis under high glucose conditions
Source: Sci Rep. 2023 Oct 2;13:16550. doi: 10.1038/s41598-023-43786-4 (PMC10545827; doi:10.1038/s41598-023-43786-4)

Figure S1

Full length and uncropped blots of eNOS, iNOS and GAPDH for Figure 3D. Red boxes indicate areas that were cropped.

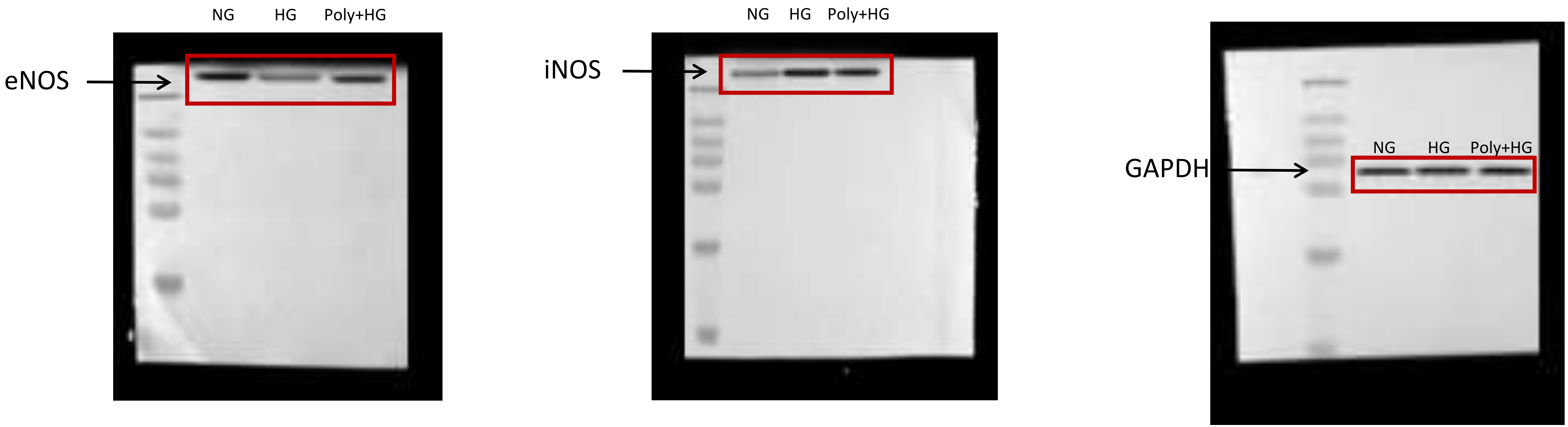

Figure S2

Full length and uncropped blots of NLRP3, VCAM-1 and GAPDH for Figure 5A, 5C. Red boxes indicate areas that were cropped.

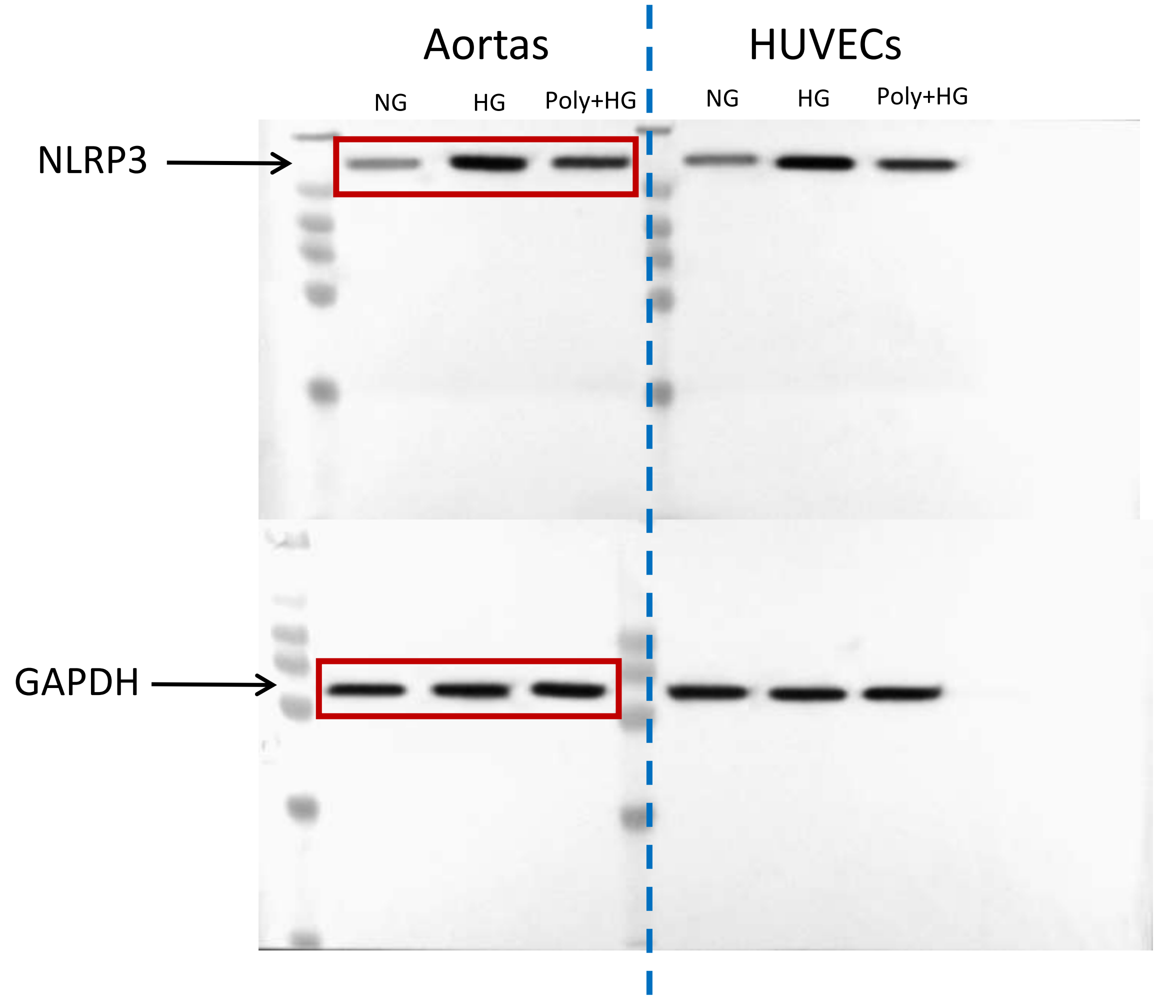

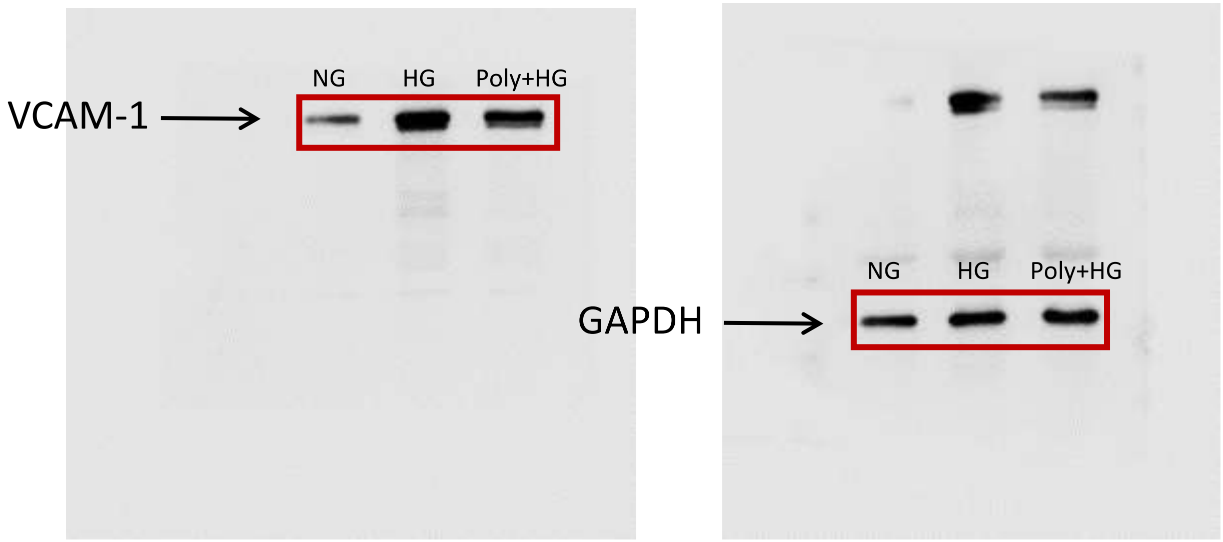

Figure S3

Full length and uncropped blots of Caspase1 and GAPDH for Figure 5E. Red boxes indicate areas that were cropped.

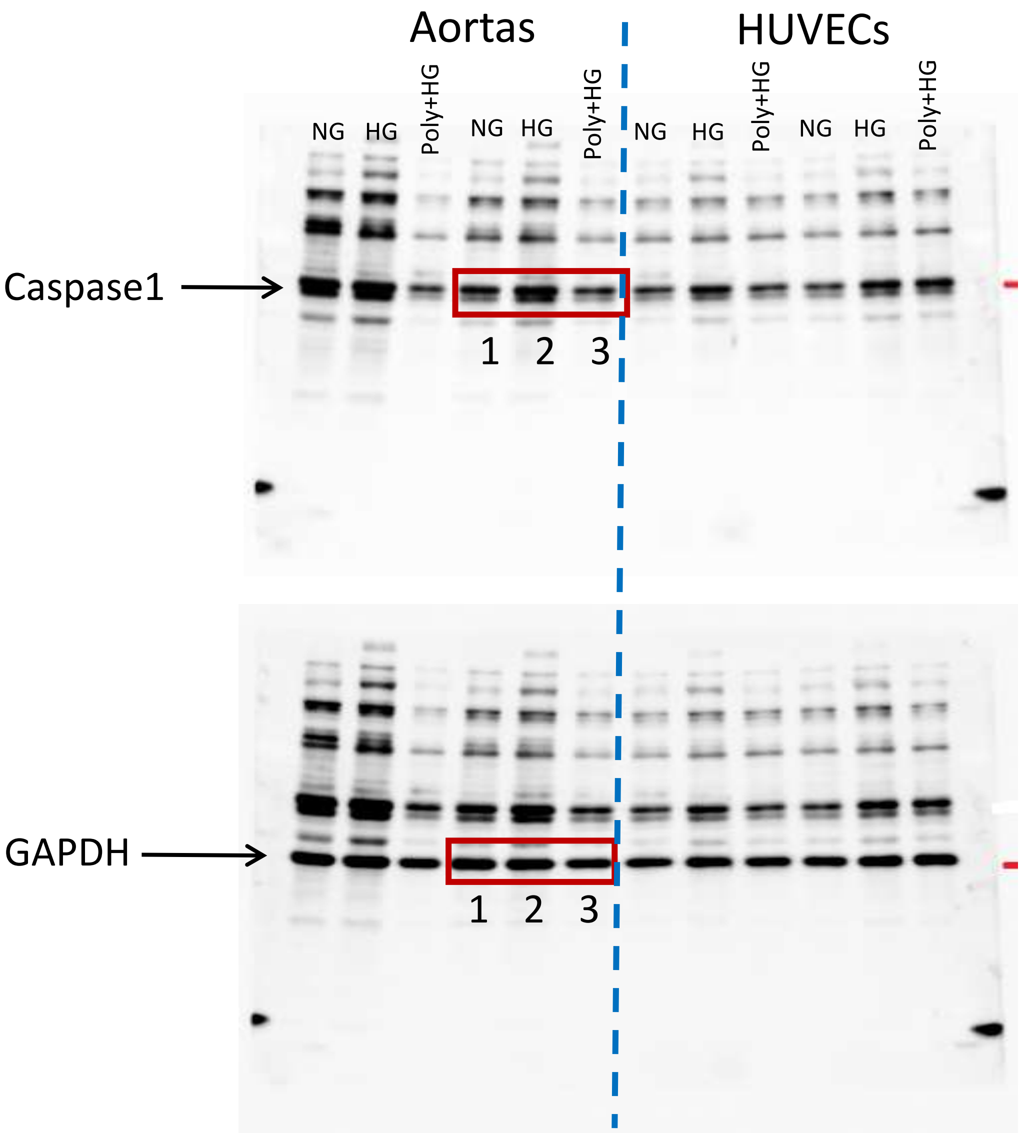

Figure S4

Full length and uncropped blots of IL-1β and GAPDH for Figure 5G. Red boxes indicate areas that were cropped in Figure 5G.

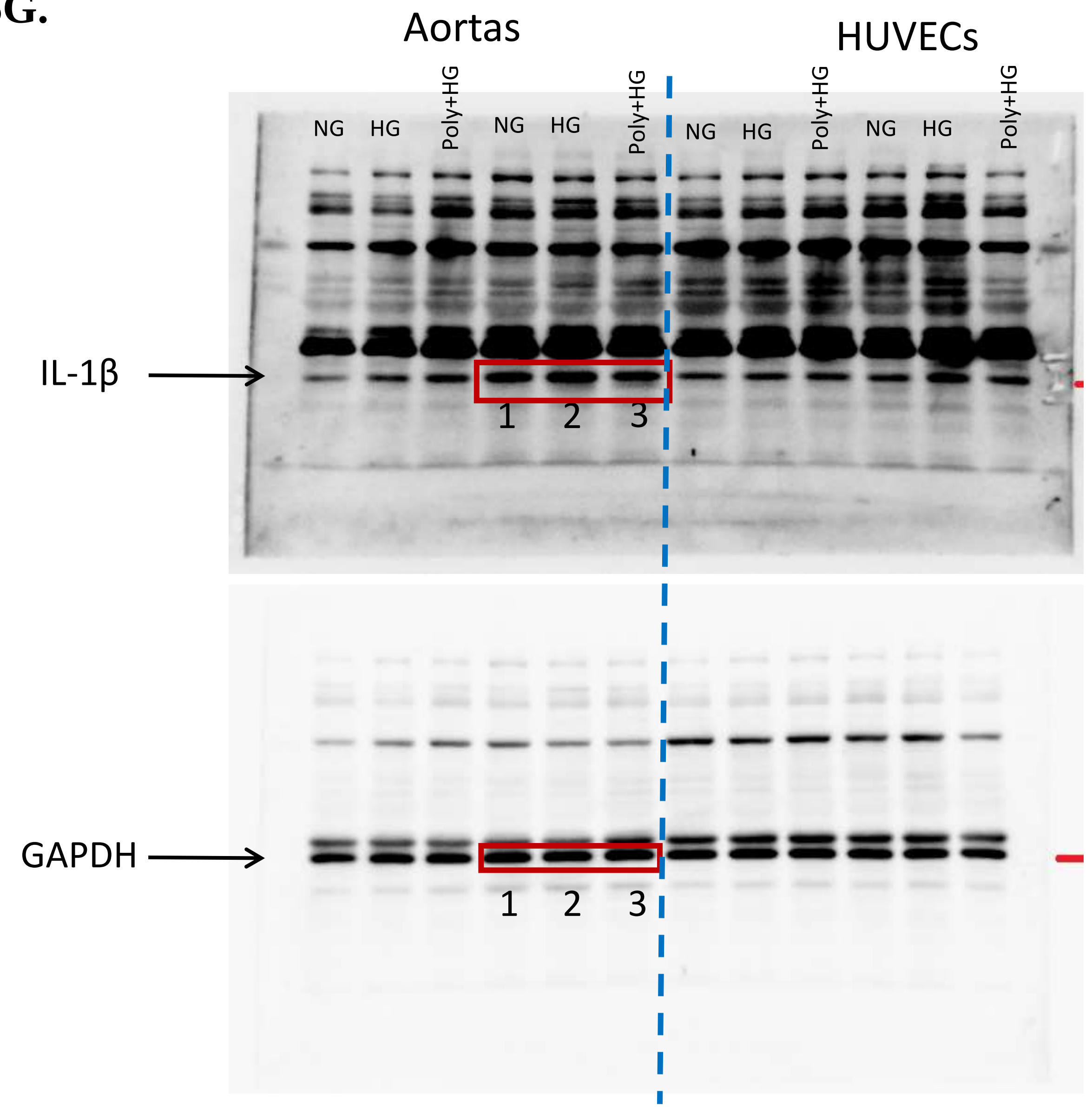

Figure S5

Full length and uncropped blots of NLRP3, VCAM-1 and GAPDH for Figure 6A, 6C. Red boxes indicate areas that were cropped.

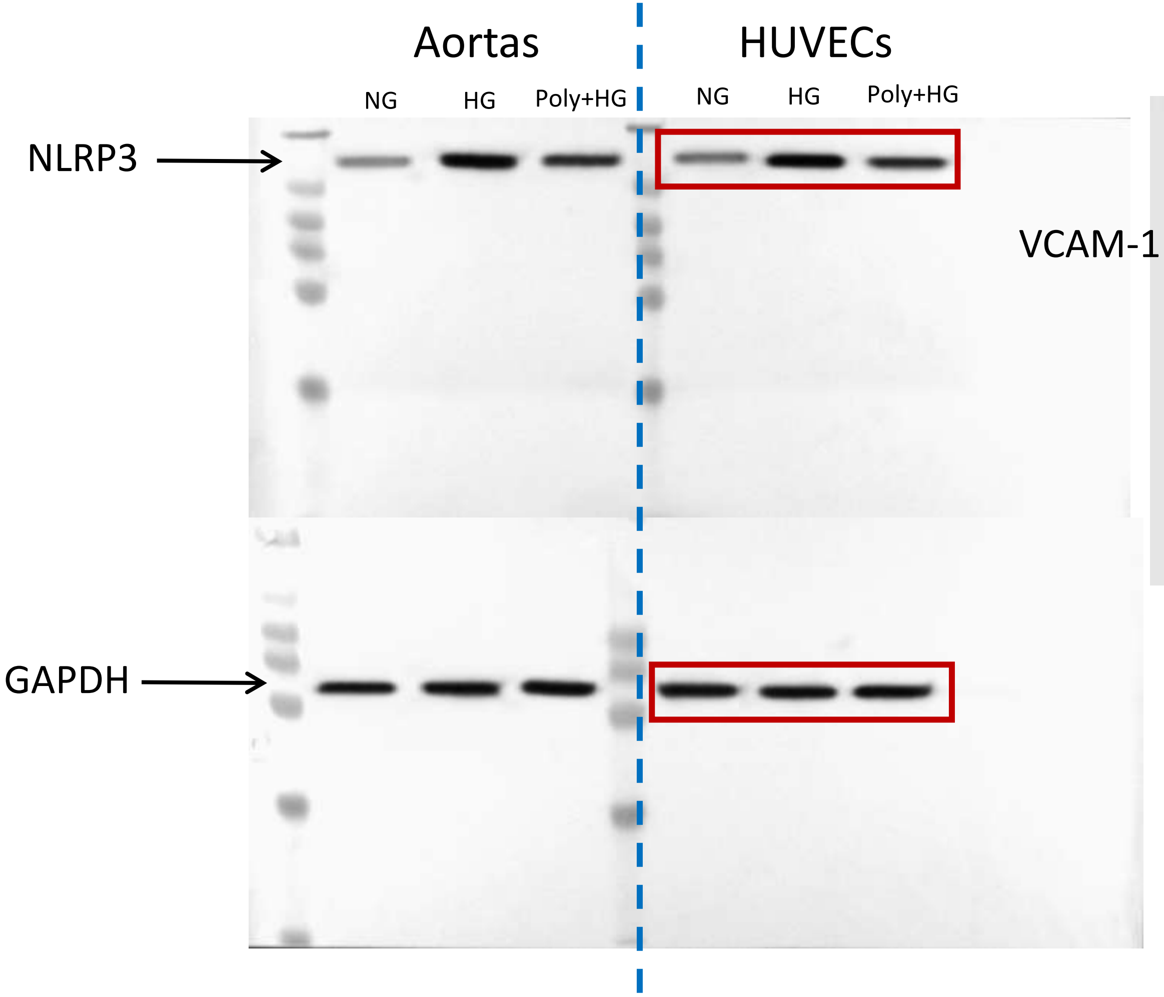

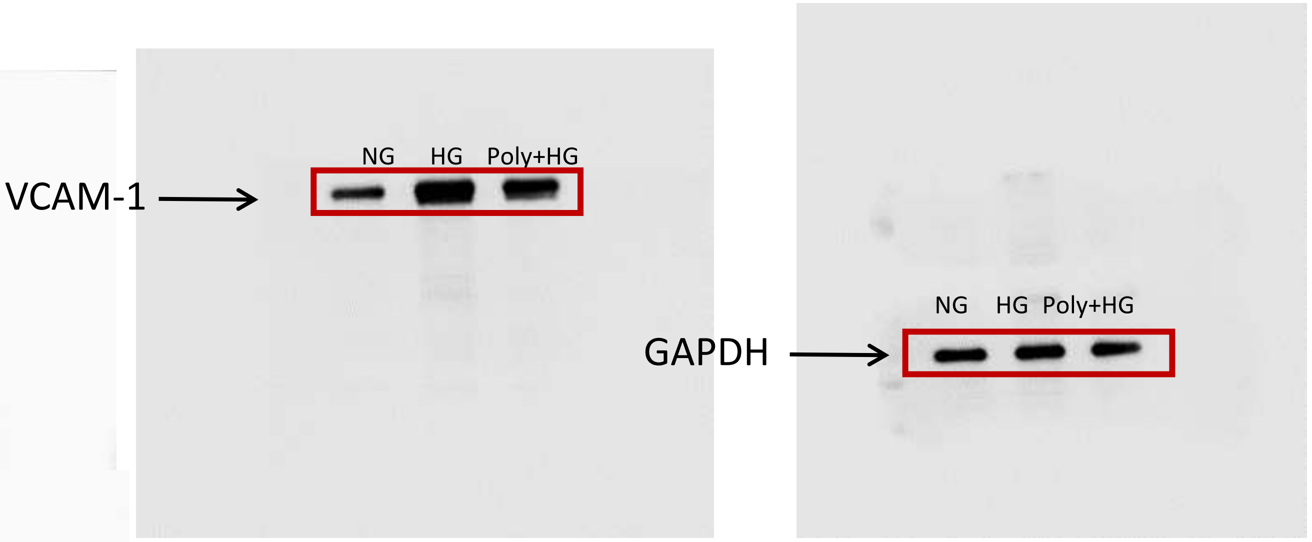

Figure S6

Full length and uncropped blots of Caspase1 and GAPDH for Figure 6E. Red boxes indicate areas that were cropped.

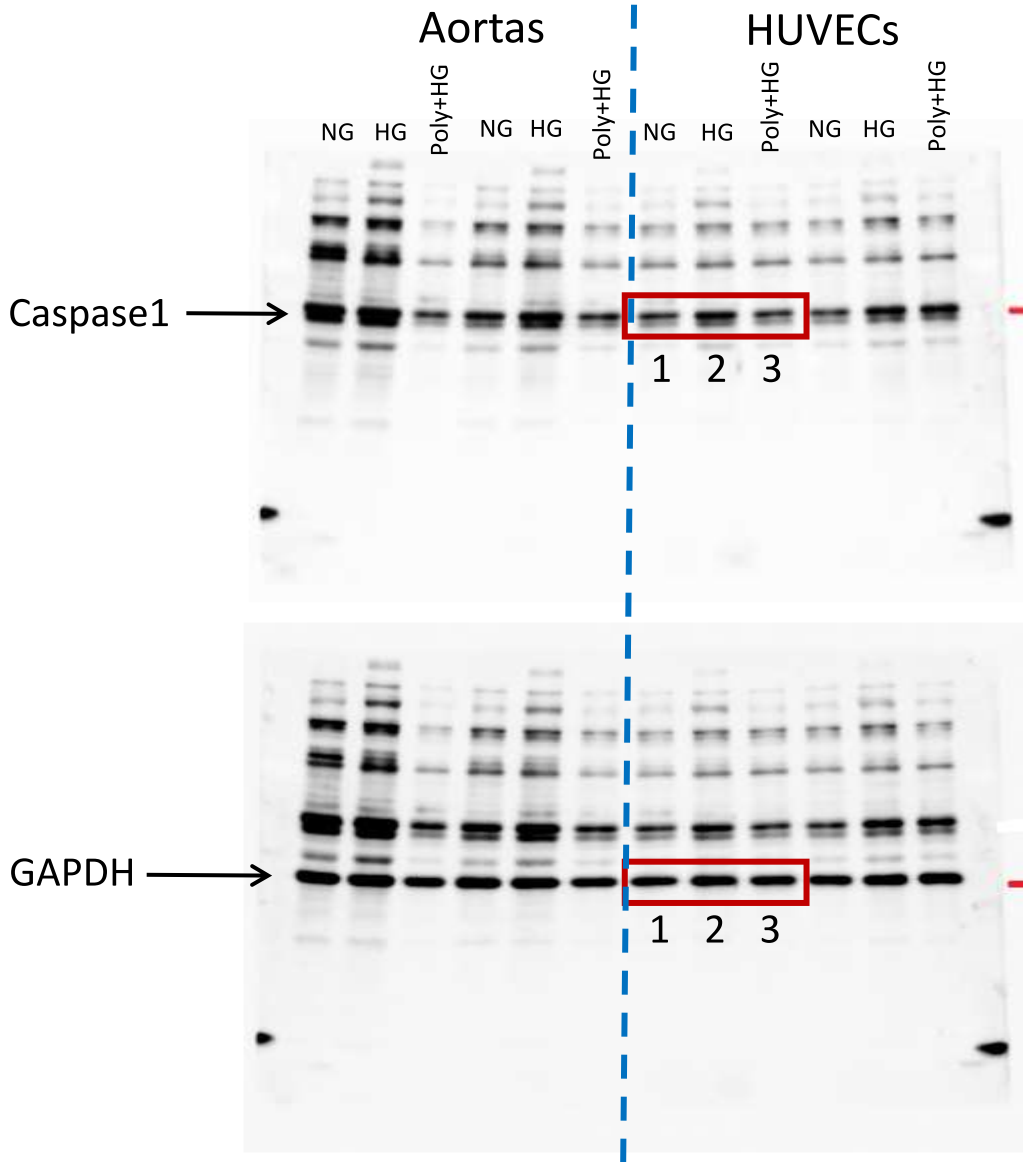

Figure S7

Full length and uncropped blots of IL-1β and GAPDH for Figure 6G. Red boxes indicate areas that were cropped.

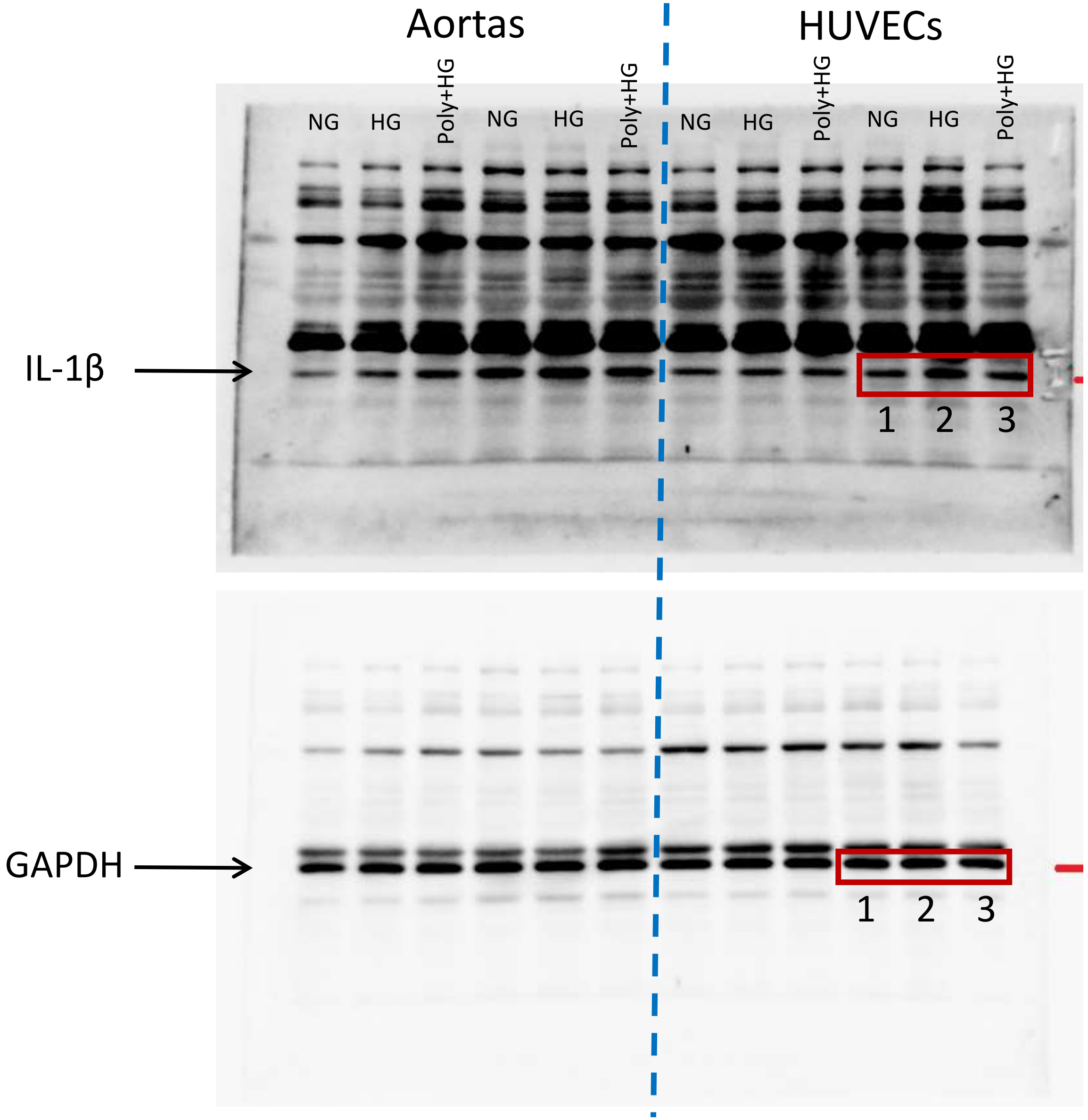

Figure S8

Full length and uncropped blots of p-Drp1, Drp1 and GAPDH for Figure 8A. Red boxes indicate areas that were cropped.

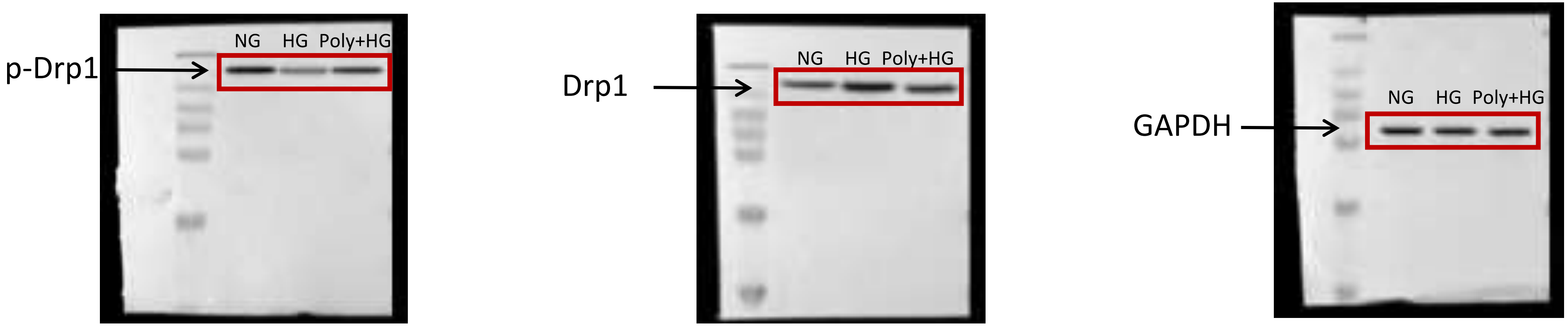

Figure S9

Full length and uncropped blots of FIS1 and GAPDH for Figure 8E. Red boxes indicate areas that were cropped.

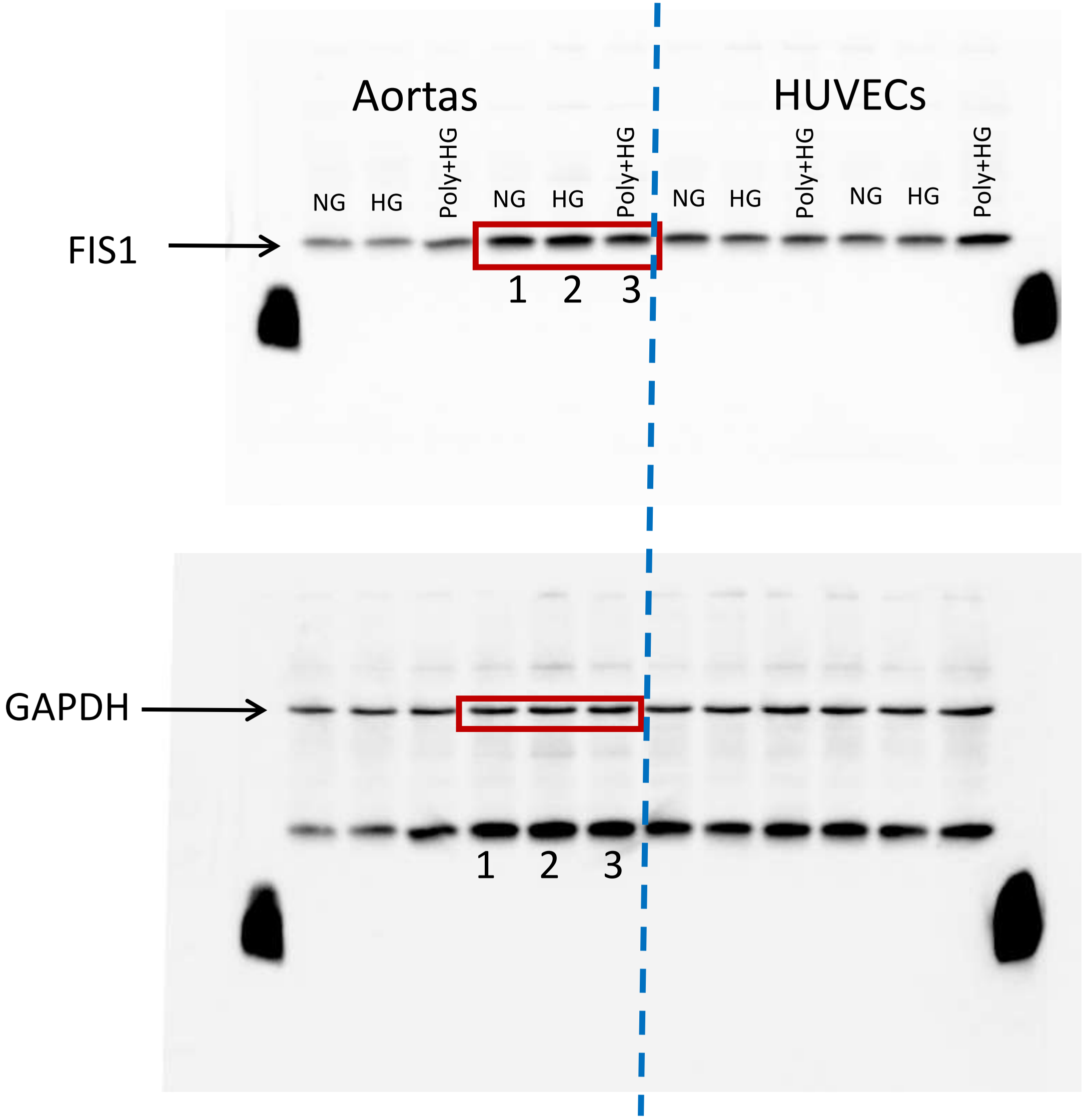

Figure S10

Full length and uncropped blots of MFN1 and GAPDH for Figure 8G. Red boxes indicate areas that were cropped.

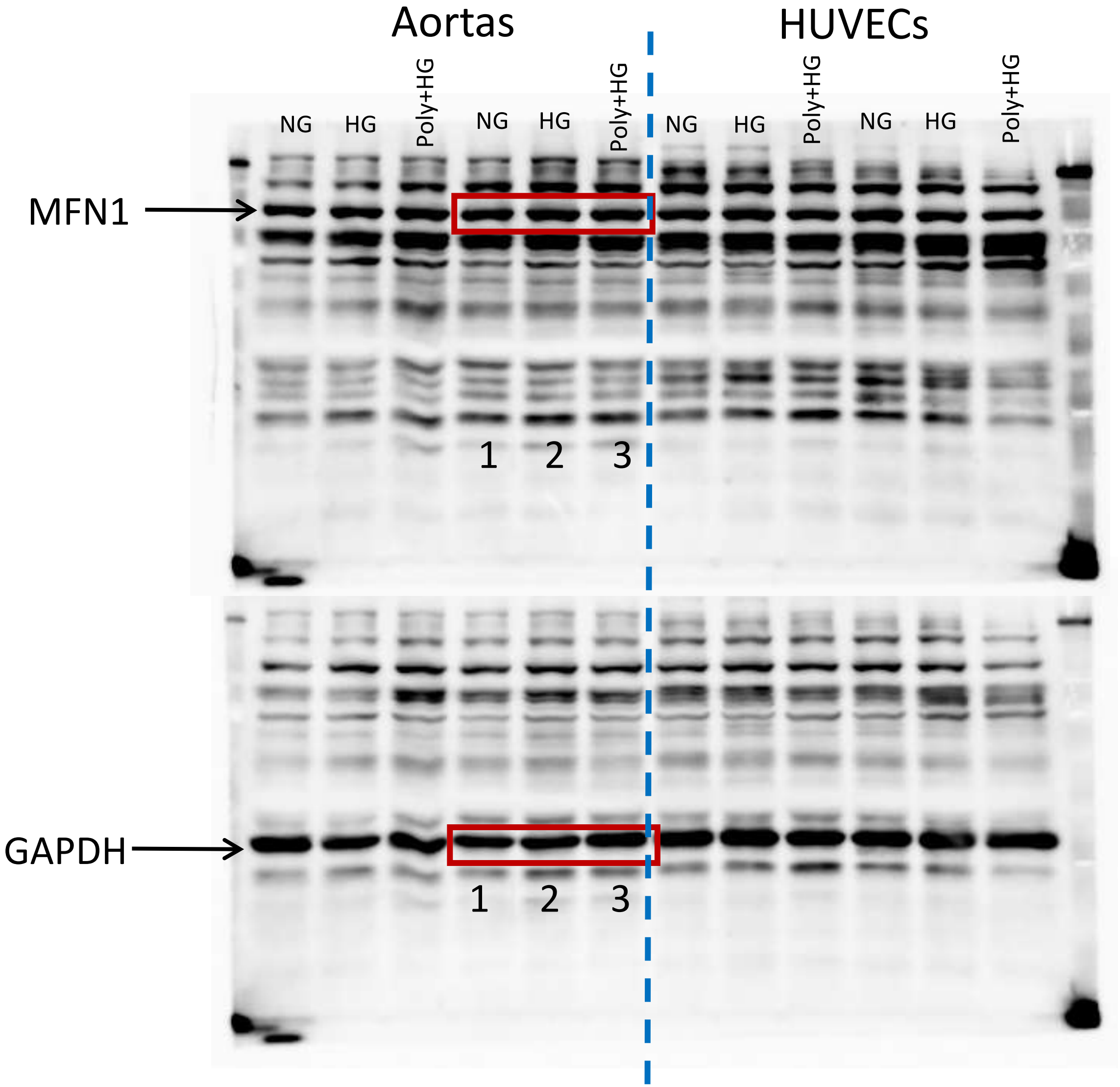

Figure S11

Full length and uncropped blots of MFN2 and GAPDH for Figure 8I. Red boxes indicate areas that were cropped.

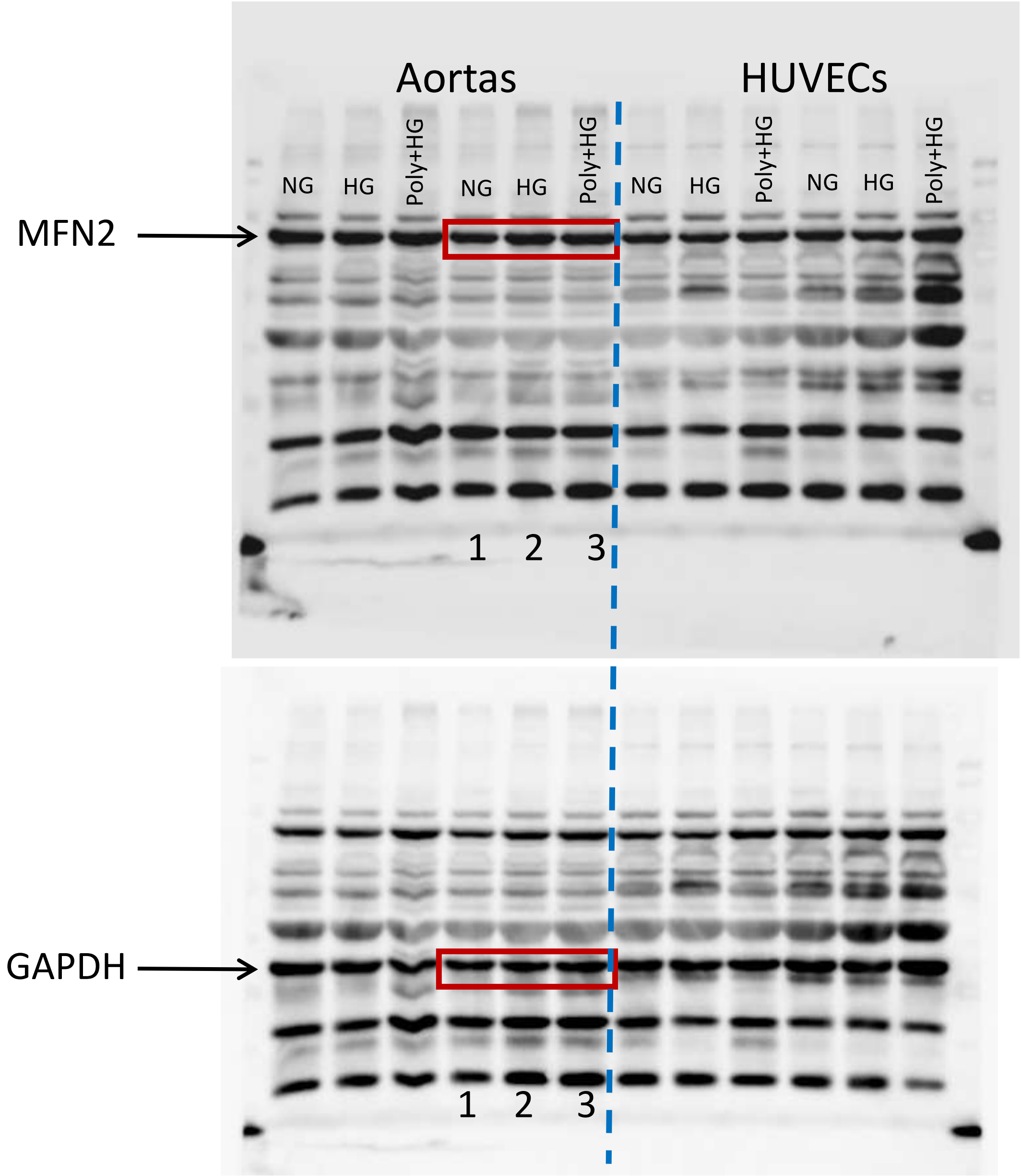

Figure S12

Full length and uncropped blots of OPA1 and GAPDH for Figure 8K. Red boxes indicate areas that were cropped.

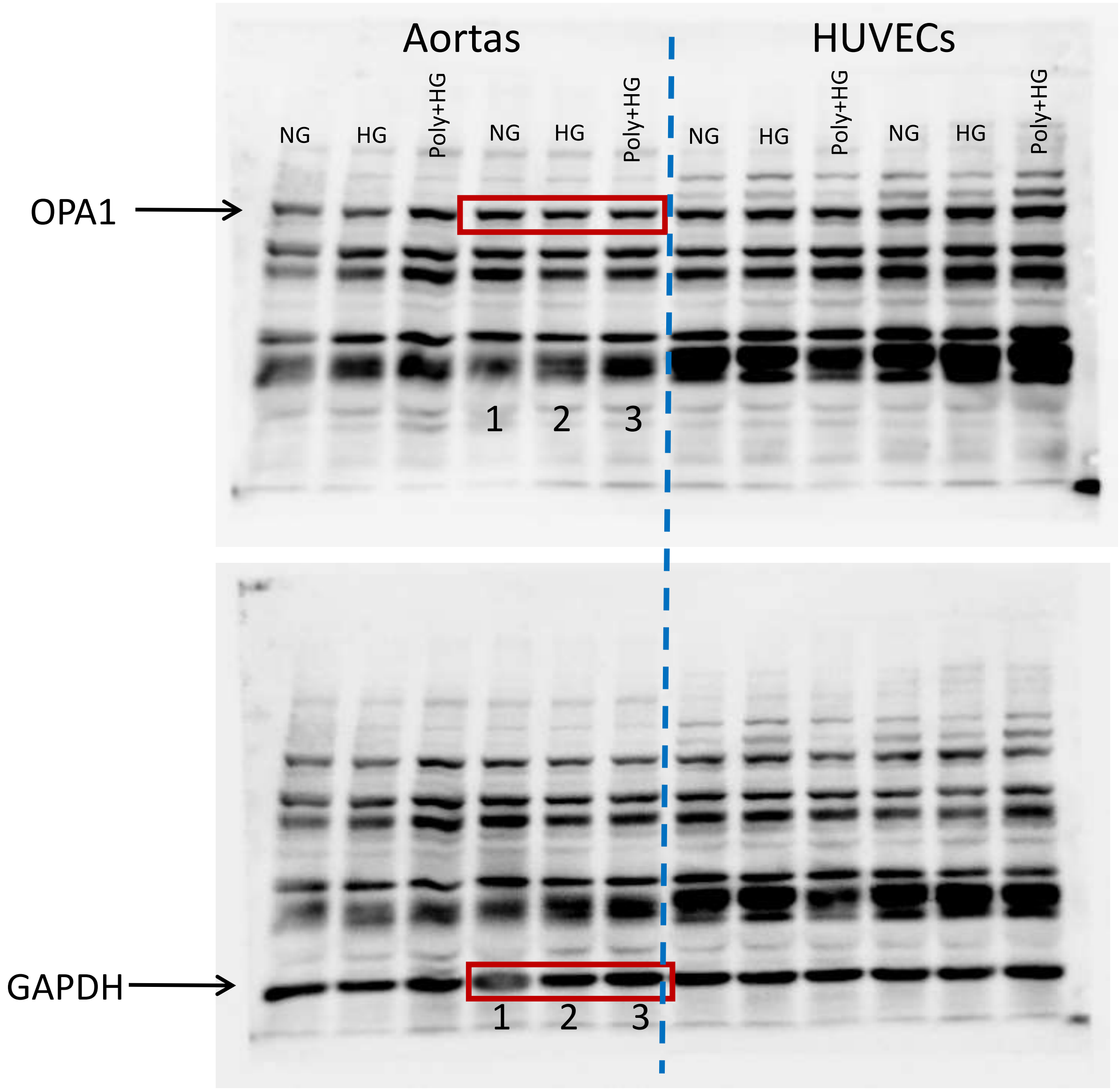

Figure S13

Full length and uncropped blots of p-Drp1, Drp1 and GAPDH for Figure 9A. Red boxes indicate areas that were cropped.

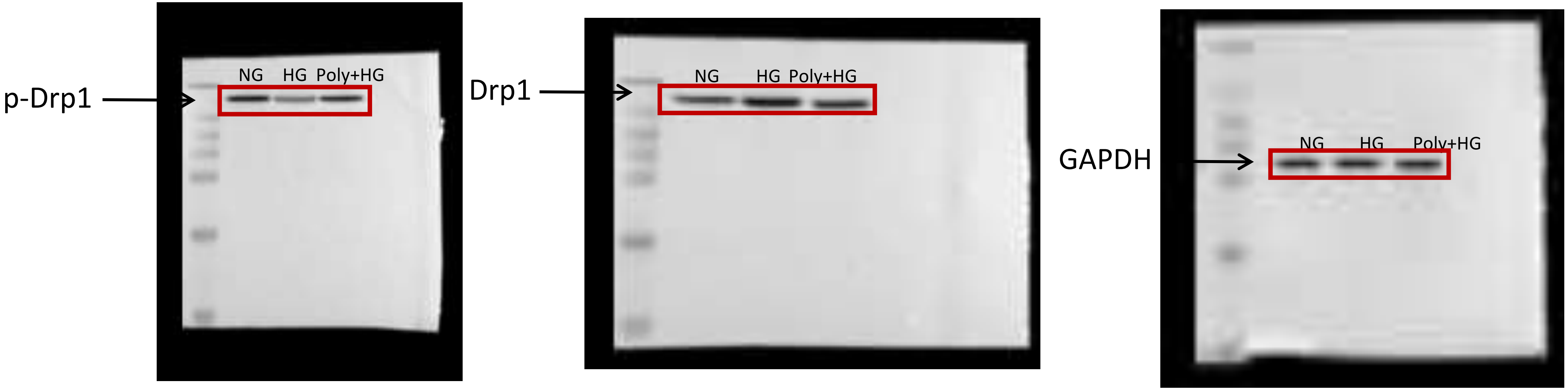

Figure S14

Full length and uncropped blots of FIS1and GAPDH for Figure 9E. Red boxes indicate areas that were cropped.

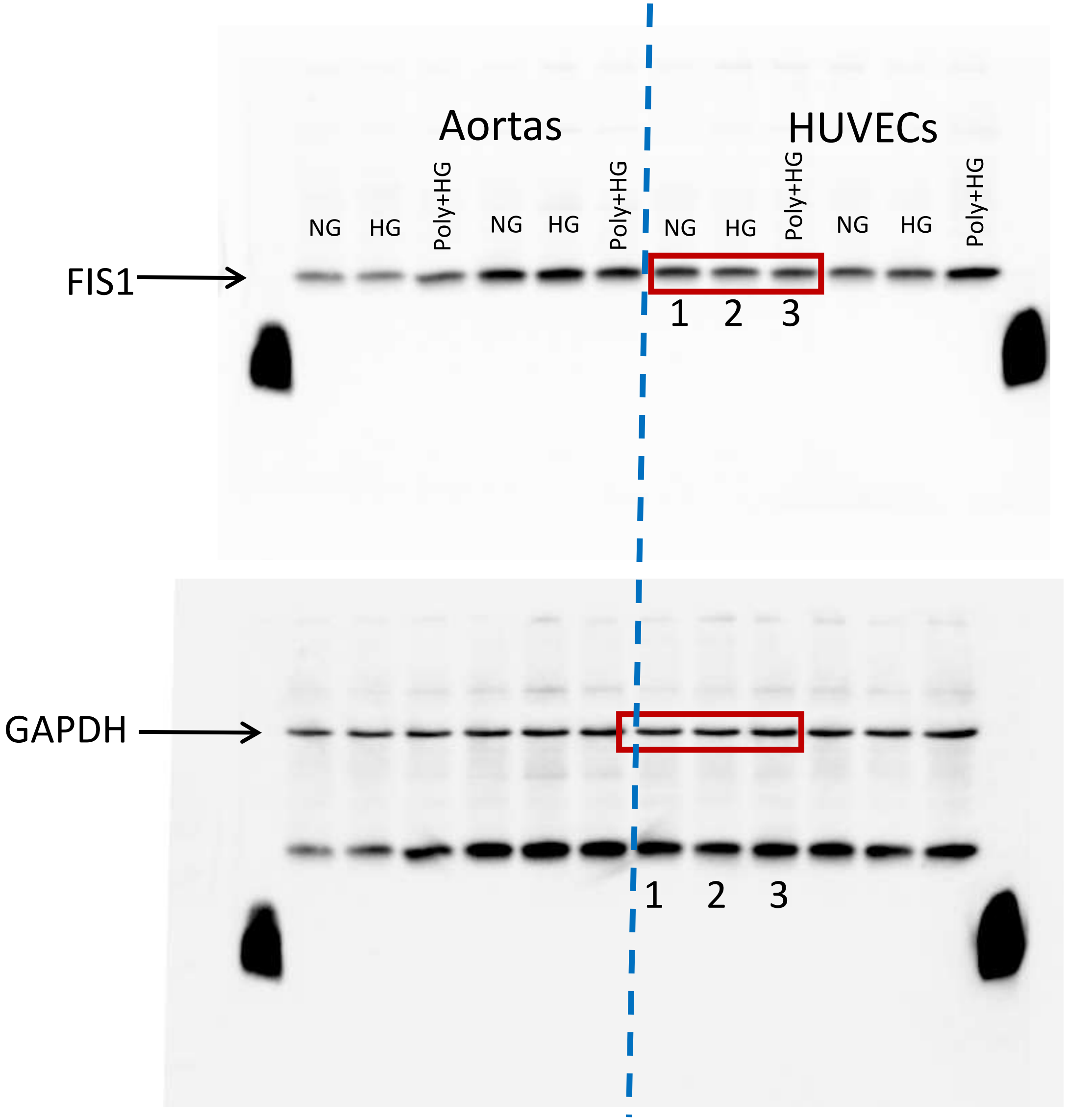

Figure S15

Full length and uncropped blots of MFN1 and GAPDH for Figure 9G. Red boxes indicate areas that were cropped.

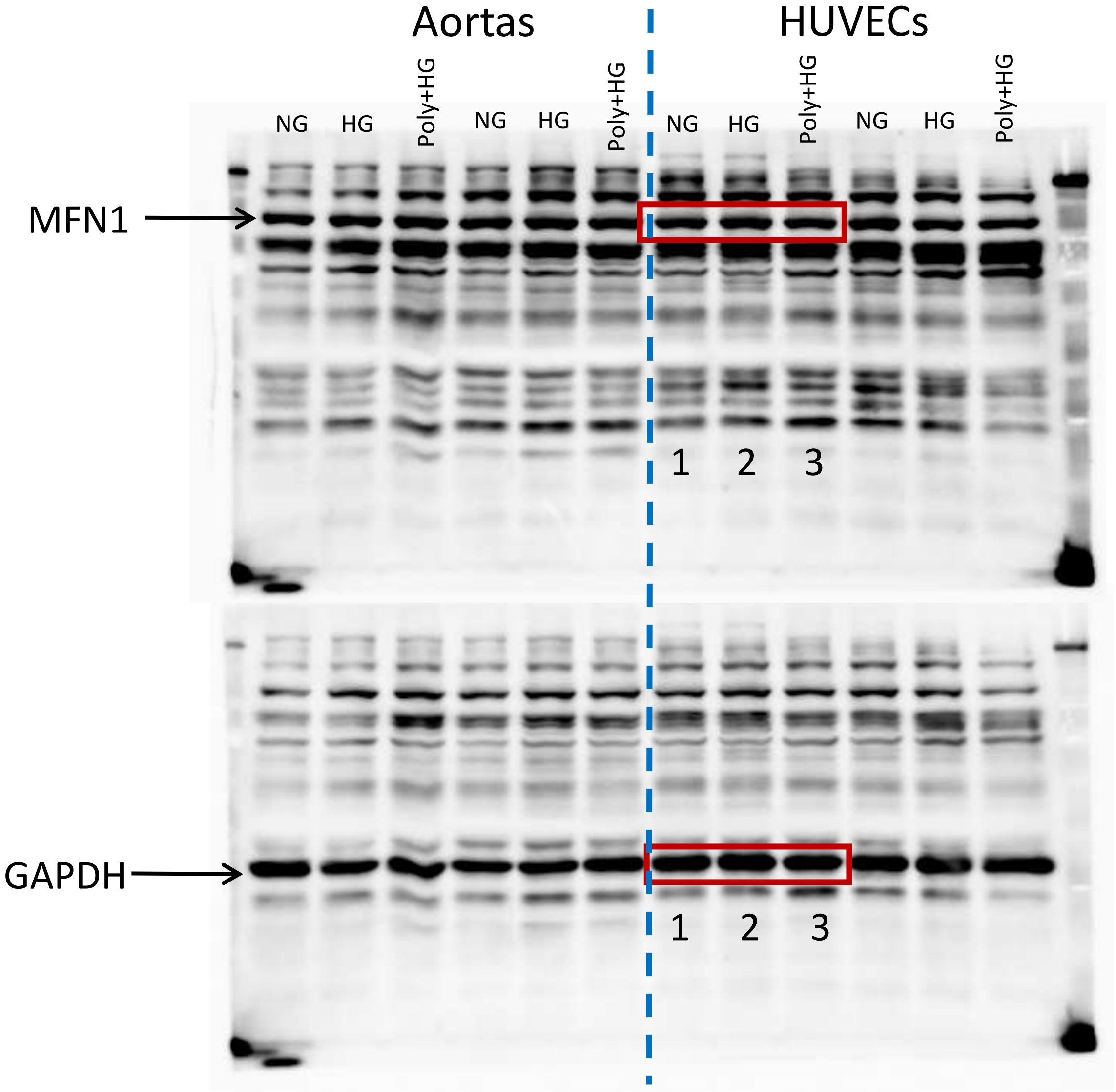

Figure S16

Full length and uncropped blots of MFN2 and GAPDH for Figure 9I. Red boxes indicate areas that were cropped.

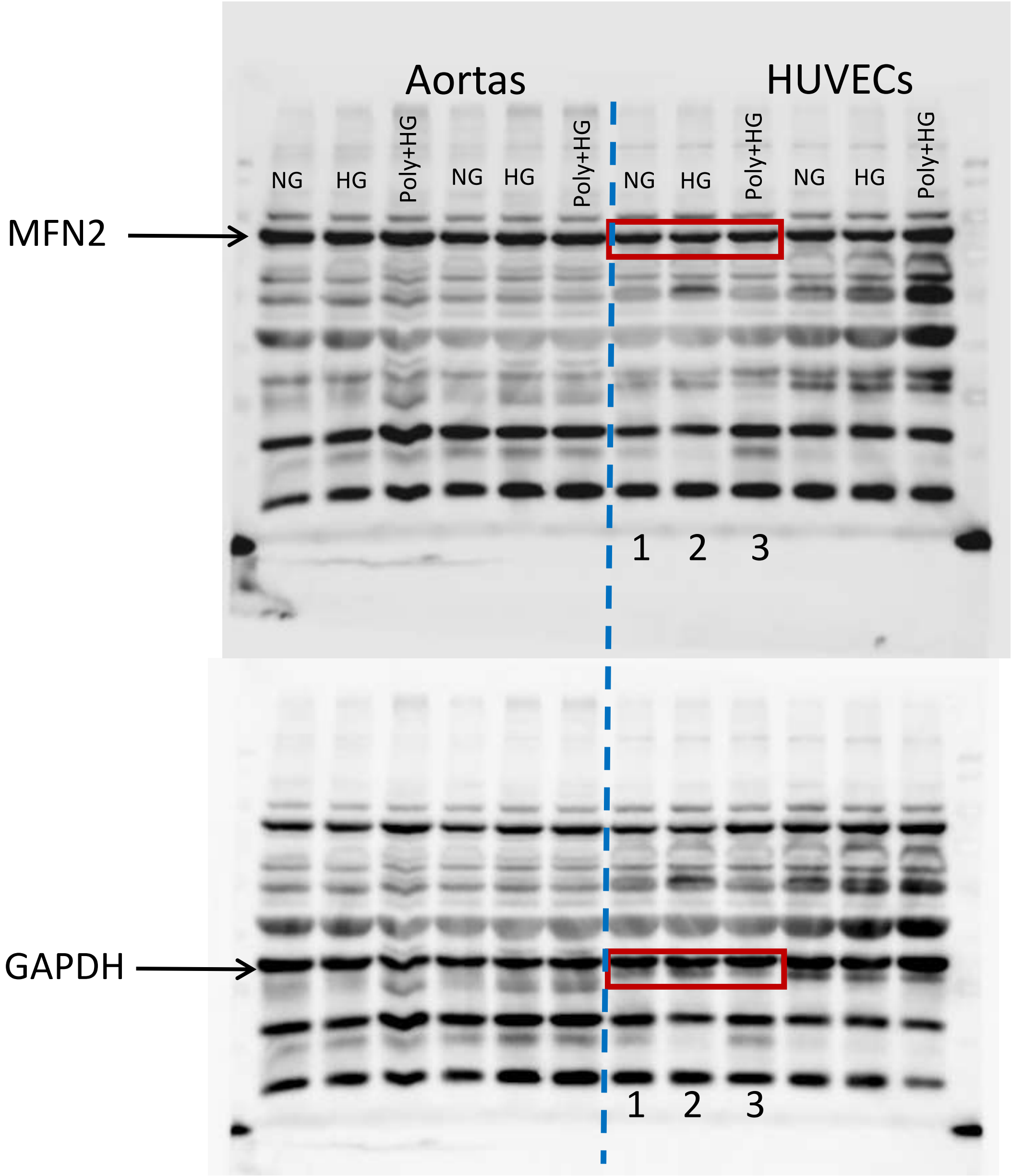

Figure S17

Full length and uncropped blots of OPA1 and GAPDH for Figure 9K. Red boxes indicate areas that were cropped.

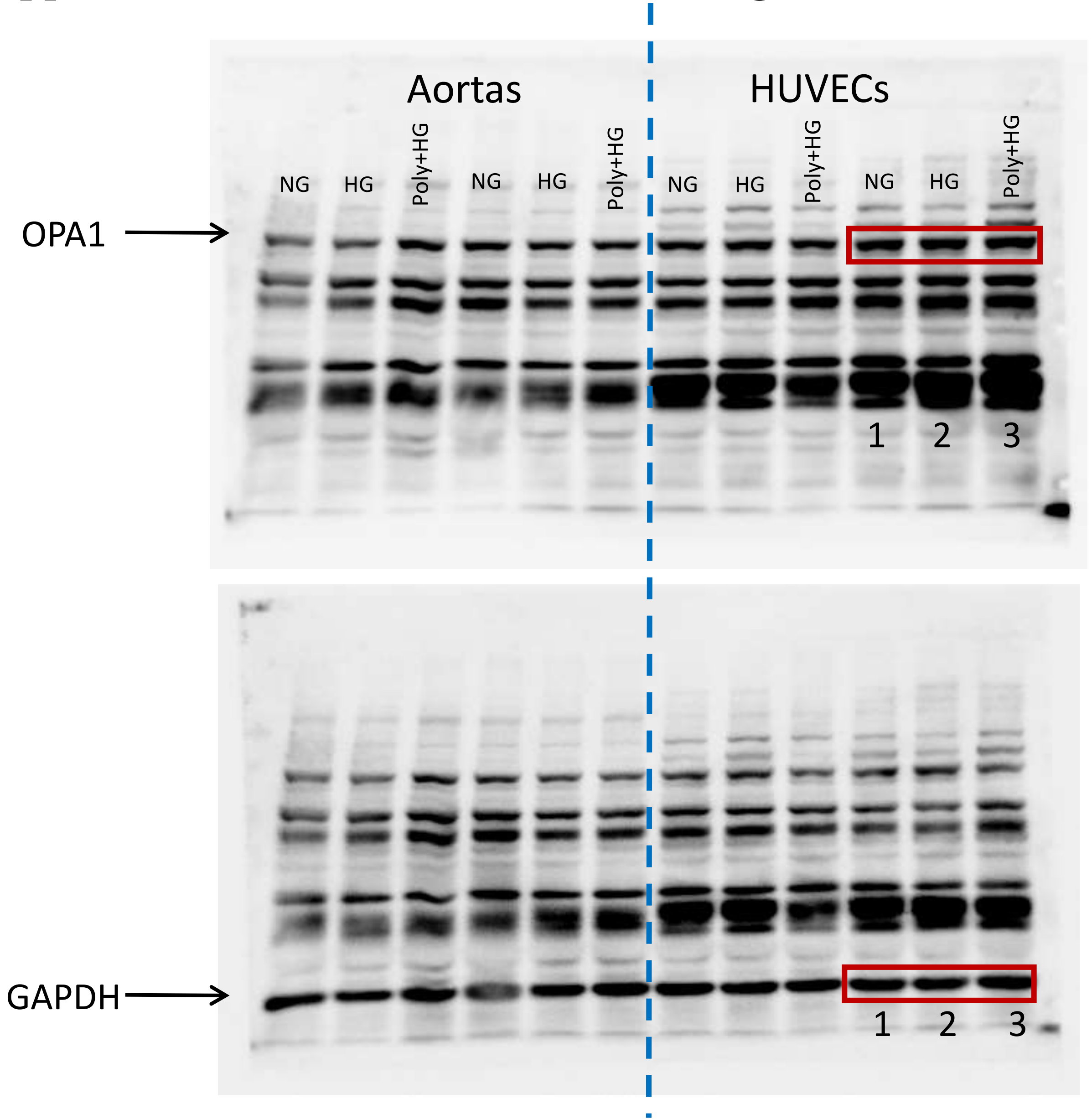

Supplement: Supplementary file 1 — Supplementary Information 1. [file 41598_2023_43786_MOESM1_ESM.pdf]
