# Supplementary material for: Polydatin improves vascular endothelial function by maintaining mitochondrial homeostasis under high glucose conditions
Source: Sci Rep. 2023 Oct 2;13:16550. doi: 10.1038/s41598-023-43786-4 (PMC10545827; doi:10.1038/s41598-023-43786-4)
Supplement: Supplementary file 2 — Supplementary Information 2. [file 41598_2023_43786_MOESM2_ESM.pptx]

## Slide 1
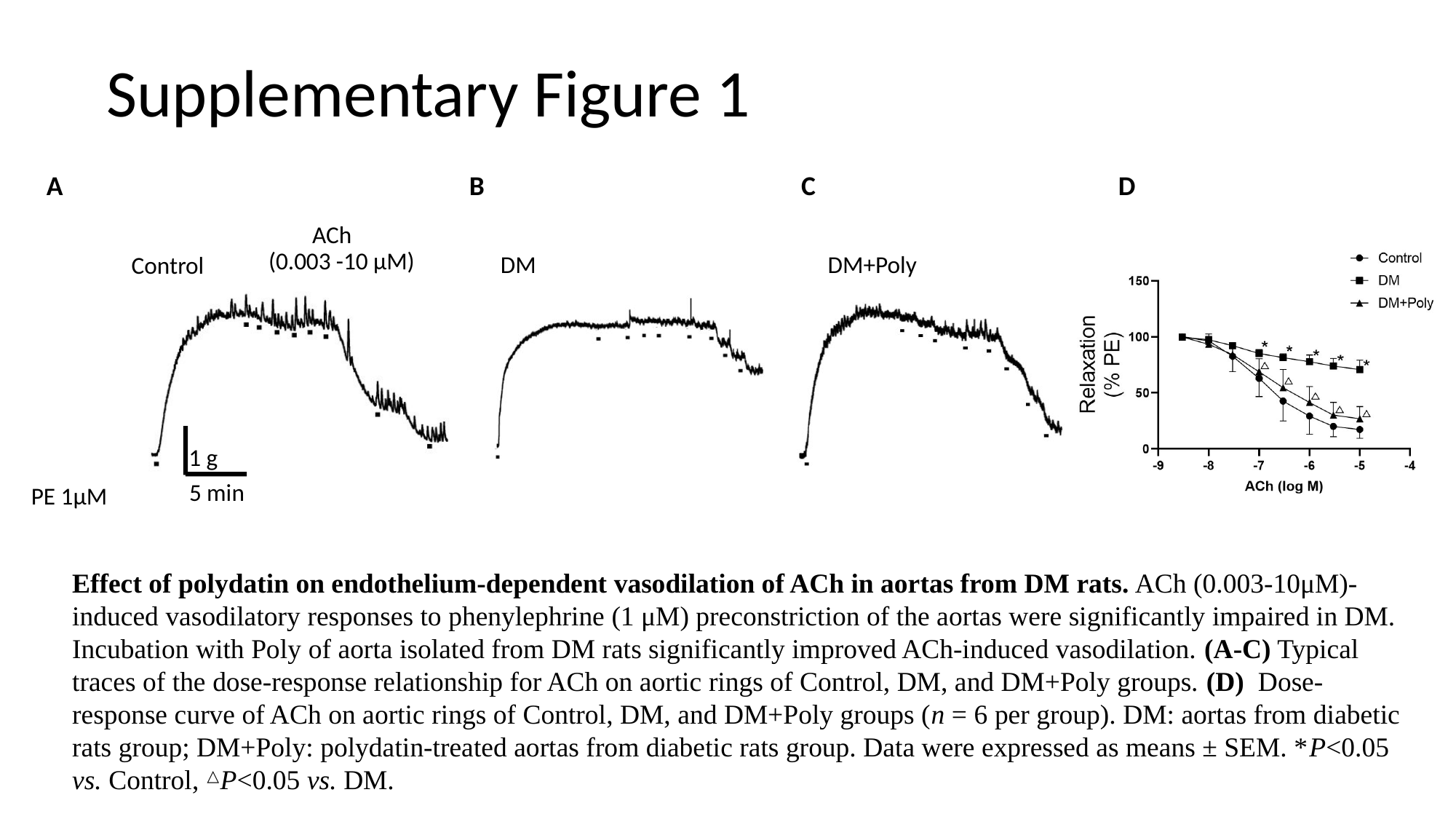

# Supplementary Figure 1
A
B
C
D
 ACh
(0.003 -10 µM)
DM
DM+Poly
Control
1 g
5 min
PE 1μM
Effect of polydatin on endothelium-dependent vasodilation of ACh in aortas from DM rats. ACh (0.003-10μM)-induced vasodilatory responses to phenylephrine (1 μM) preconstriction of the aortas were significantly impaired in DM. Incubation with Poly of aorta isolated from DM rats significantly improved ACh-induced vasodilation. (A-C) Typical traces of the dose-response relationship for ACh on aortic rings of Control, DM, and DM+Poly groups. (D) Dose-response curve of ACh on aortic rings of Control, DM, and DM+Poly groups (n = 6 per group). DM: aortas from diabetic rats group; DM+Poly: polydatin-treated aortas from diabetic rats group. Data were expressed as means ± SEM. *P<0.05 vs. Control, △P<0.05 vs. DM.

## Slide 2
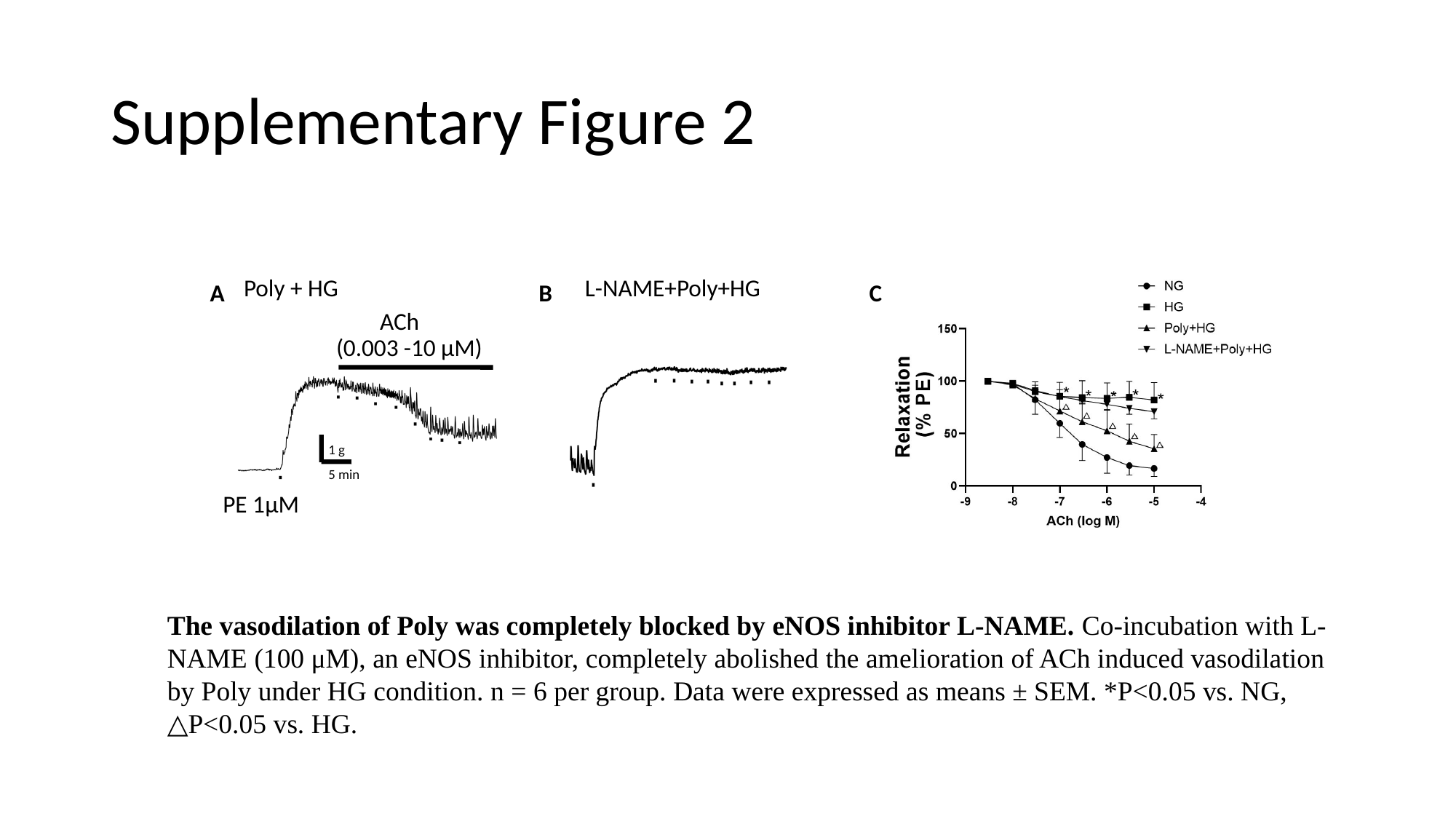

# Supplementary Figure 2
A
Poly + HG
B
L-NAME+Poly+HG
C
 ACh
(0.003 -10 µM)
1 g
5 min
PE 1μM
The vasodilation of Poly was completely blocked by eNOS inhibitor L-NAME. Co-incubation with L-NAME (100 μM), an eNOS inhibitor, completely abolished the amelioration of ACh induced vasodilation by Poly under HG condition. n = 6 per group. Data were expressed as means ± SEM. *P<0.05 vs. NG, △P<0.05 vs. HG.

## Slide 3
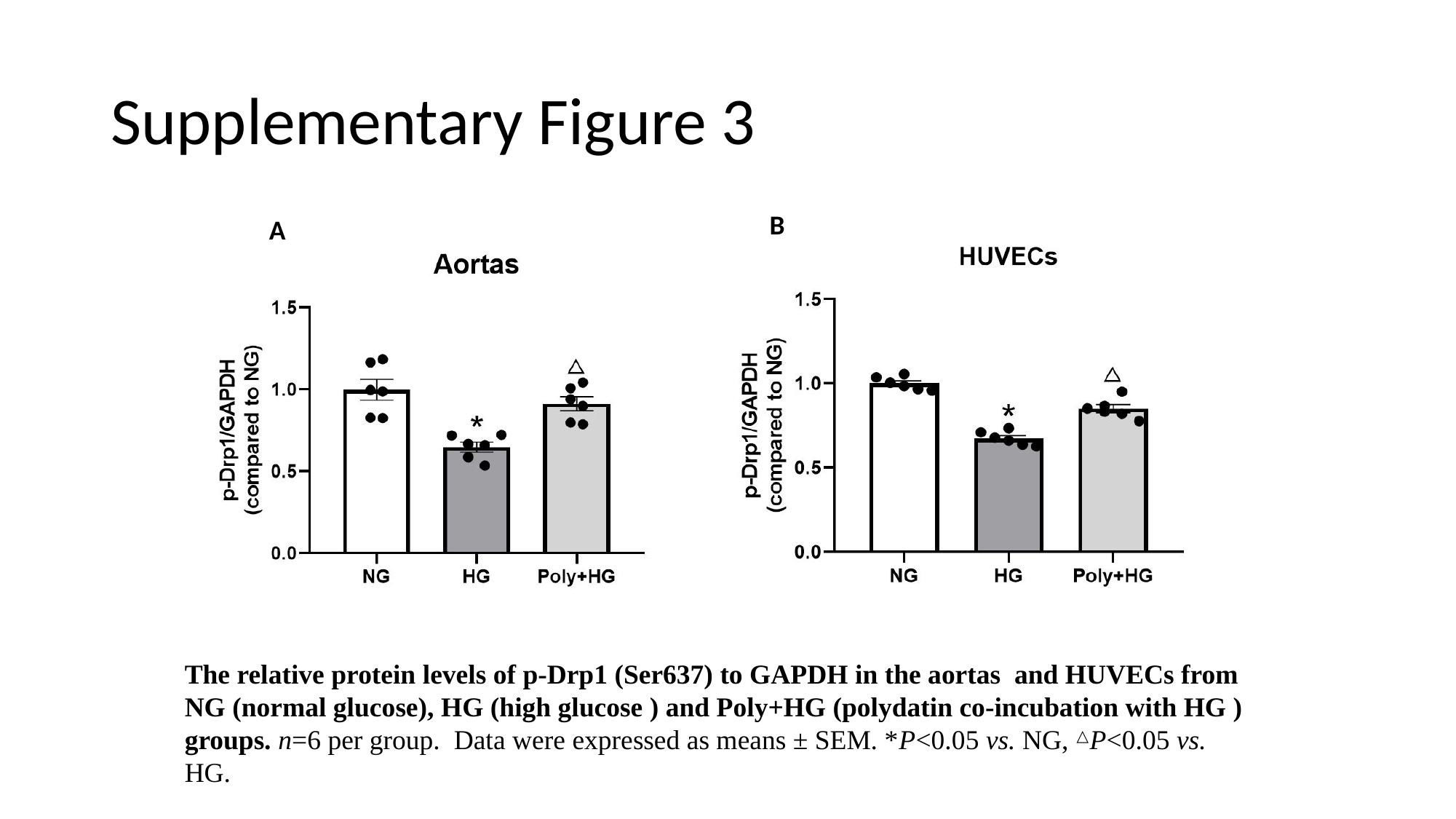

# Supplementary Figure 3
B
A
The relative protein levels of p-Drp1 (Ser637) to GAPDH in the aortas and HUVECs from NG (normal glucose), HG (high glucose ) and Poly+HG (polydatin co-incubation with HG ) groups. n=6 per group. Data were expressed as means ± SEM. *P<0.05 vs. NG, △P<0.05 vs. HG.

## Slide 4
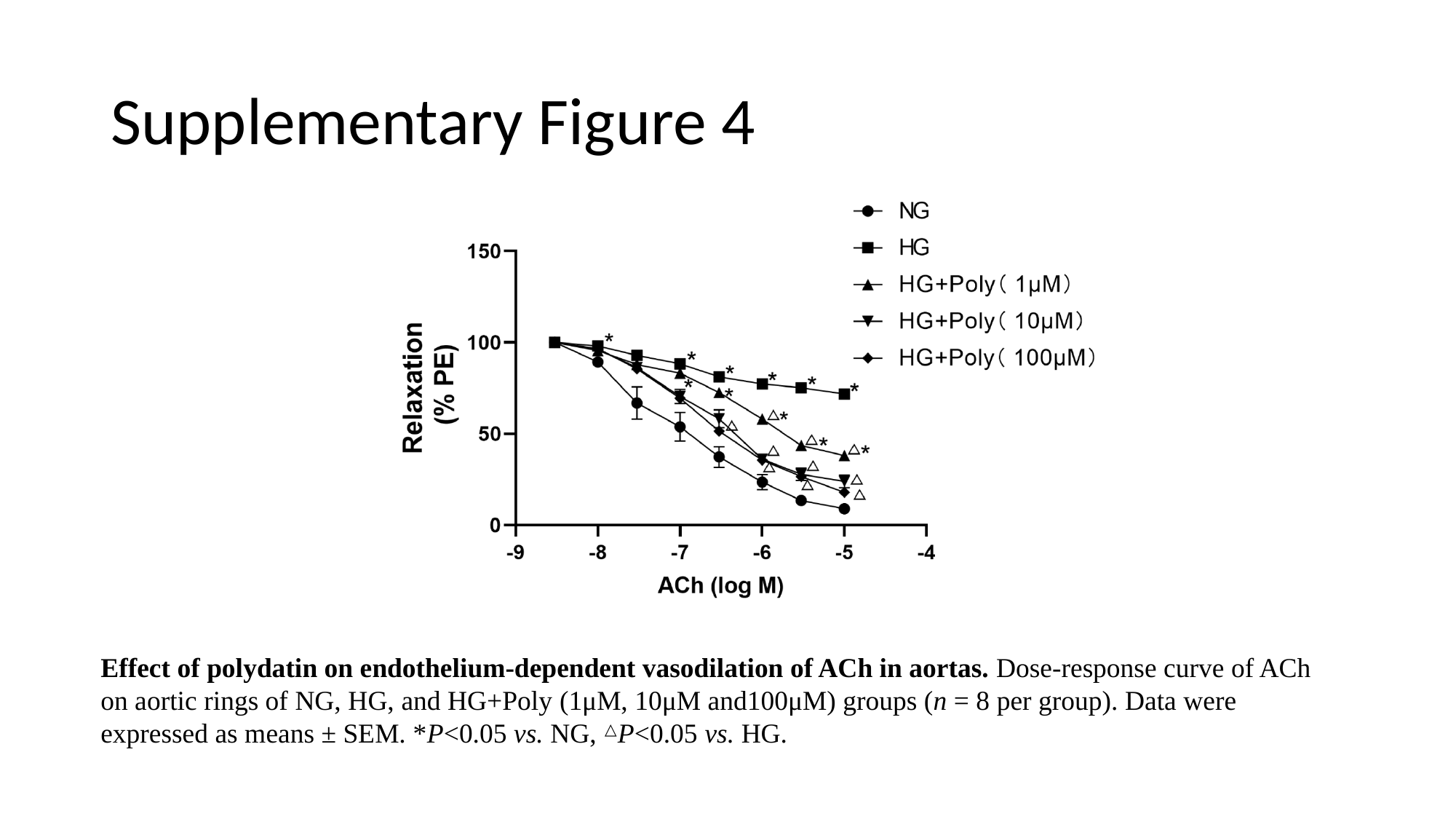

# Supplementary Figure 4
Effect of polydatin on endothelium-dependent vasodilation of ACh in aortas. Dose-response curve of ACh on aortic rings of NG, HG, and HG+Poly (1μM, 10μM and100μM) groups (n = 8 per group). Data were expressed as means ± SEM. *P<0.05 vs. NG, △P<0.05 vs. HG.

## Slide 5
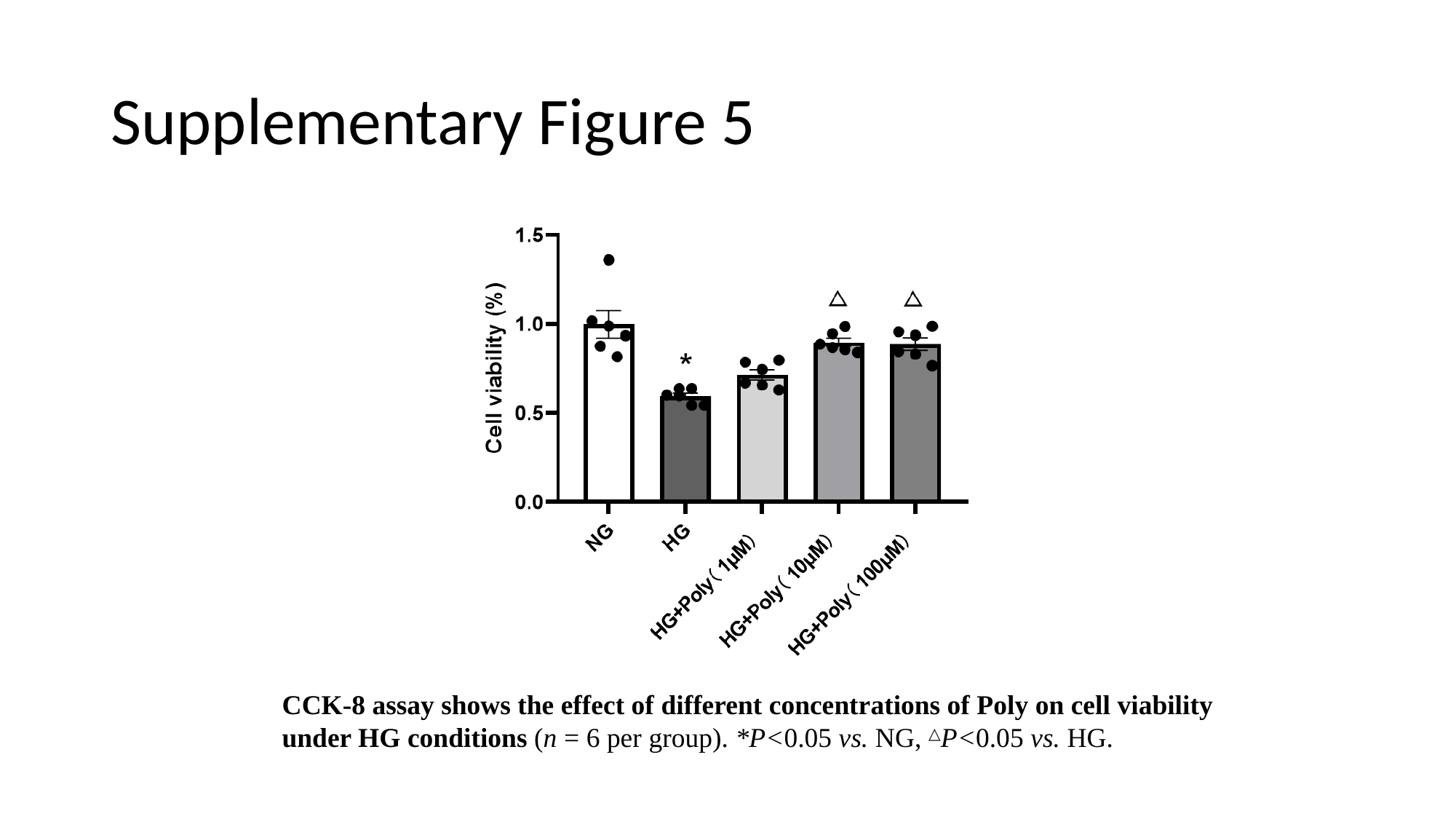

# Supplementary Figure 5
CCK-8 assay shows the effect of different concentrations of Poly on cell viability under HG conditions (n = 6 per group). *P<0.05 vs. NG, △P<0.05 vs. HG.
